# Particulate Matter Exposure and Diabetic Kidney Dysfunction: Insights from Integrated Transcriptomic and Bioinformatics Analyses

**DOI:** 10.3390/ijms27177615

**Published:** 2026-08-25

**Authors:** Jiang Tan, Yuqin Chen, Jiliang Hu

**Affiliations:** 1 College of Artificial Intelligence Medicine, Chongqing Medical University, No.1 of Yixueyuan Road, Yu Zhong District, Chongqing 400016, China; 2 Center for Neuroscience, College of Basic Medicine, Chongqing Medical University, Chongqing 400016, China

**Keywords:** particulate matter, diabetic kidney disease, machine learning, air pollution

## Abstract

Exposure to ambient particulate matter (PM) has been linked to renal dysfunction, particularly in diabetic populations, but the underlying mechanisms remain unclear. We performed bidirectional Mendelian randomization to assess causal relationships between PM exposure and estimated glomerular filtration rate (eGFR), integrated transcriptomic datasets to identify PM-related genes overlapping with diabetic kidney disease (DKD) differentially expressed genes, and applied machine learning approaches to select key feature genes and construct diagnostic models. Single-cell and spatial transcriptomic analyses were used to characterize cell-type and region-specific expression patterns, while in silico knockout analysis explored potential functional associations. PM2.5–10 exposure was causally associated with decreased eGFR, particularly among individuals with diabetes, with no evidence of reverse causality. Transcriptomic integration identified 168 shared PM-DKD genes enriched in inflammatory, immune, and metabolic pathways, including AGE-RAGE, IL-17, TNF, and PI3K-Akt signaling. Seven feature genes (AVPI1, DUSP1, FOSB, JUNB, PDK2, TPPP3, and VIM) showed good diagnostic performance across training and external validation cohorts, and machine learning models and nomogram analyses demonstrated consistent predictive performance. Single-cell and spatial transcriptomic analyses revealed distinct cell-type and region-specific expression patterns, with VIM enriched in interstitial and fibrotic regions, TPPP3 mainly detected in podocytes, and other genes distributed across tubular or immune cell populations. In silico knockout analysis suggested potential associations of these genes with mitochondrial metabolism, oxidative stress, tubular function, and inflammatory processes. Database-based therapeutic exploration identified VIM as a potential candidate target, with sanguinarine showing favorable predicted binding affinity. Collectively, these findings suggest that PM2.5–10 exposure may contribute to DKD susceptibility through inflammatory, metabolic, and oxidative stress-related mechanisms, and provide candidate molecular markers for further investigation.

## 1. Introduction

Diabetic kidney disease (DKD) is one of the most common and severe microvascular complications of diabetes and represents a leading cause of end-stage renal disease (ESRD) [1]. Despite significant advances in glycemic control, blood pressure management, and renal-protective therapies in recent years, the incidence and progression of DKD remain inadequately controlled, suggesting a complex pathogenesis that likely involves the interplay of genetic predisposition, metabolic dysregulation, and environmental factors.

Air pollution has been recognized as an important environmental health risk factor and has been increasingly linked to the development and progression of various chronic diseases [2,3]. Particulate matter (PM) exposure is thought to exert systemic biological effects, influencing cardiovascular function, metabolic homeostasis, and renal health [4,5]. Studies have indicated that exposure to particulate matter may be associated with declines in glomerular filtration rate, increased proteinuria, and a heightened risk of chronic kidney disease [6,7].

PM exposure may contribute to kidney injury through mechanisms such as oxidative stress induction, inflammation activation, endothelial dysfunction, and the disruption of cellular metabolic homeostasis [8]. In the context of diabetes, a hyperglycemic environment may further amplify AGE-RAGE signaling, mitochondrial dysfunction, and chronic inflammation, thereby potentially increasing renal susceptibility to environmental insults. Consequently, environmental exposures may play a significant yet not fully elucidated role in the progression of DKD. Currently, genetic evidence supporting a causal relationship between air pollution and DKD is limited, and the underlying molecular mechanisms remain poorly characterized.

Mendelian randomization (MR) leverages genetic variants as instrumental variables to mitigate confounding and reverse causation bias, providing more robust evidence for potential causal relationships between exposures and disease outcomes [9]. Meanwhile, advances in high-throughput sequencing have enabled multi-omics integrative analyses, machine learning algorithms, and single-cell transcriptomics, offering novel strategies to dissect disease mechanisms at the level of cell-type specificity and molecular networks.

The present study employed bidirectional MR analyses, transcriptomics, machine learning, single-cell RNA sequencing (scRNA-Seq), spatial transcriptomics, and molecular docking approaches to systematically investigate the potential causal relationship between particulate matter exposure and renal dysfunction. Furthermore, we aimed to identify candidate molecular signatures and explore potential biological mechanisms associated with DKD progression. This study provides a comprehensive multi-omics framework for understanding the association between environmental exposure and DKD and may facilitate the identification of potential biomarkers and therapeutic candidates for further investigation.

## 2. Results

### 2.1. Particulate Matter Exposure Is Associated with Renal Function Decline, with More Pronounced Effects Observed in Diabetic Populations

**Figure 1:**
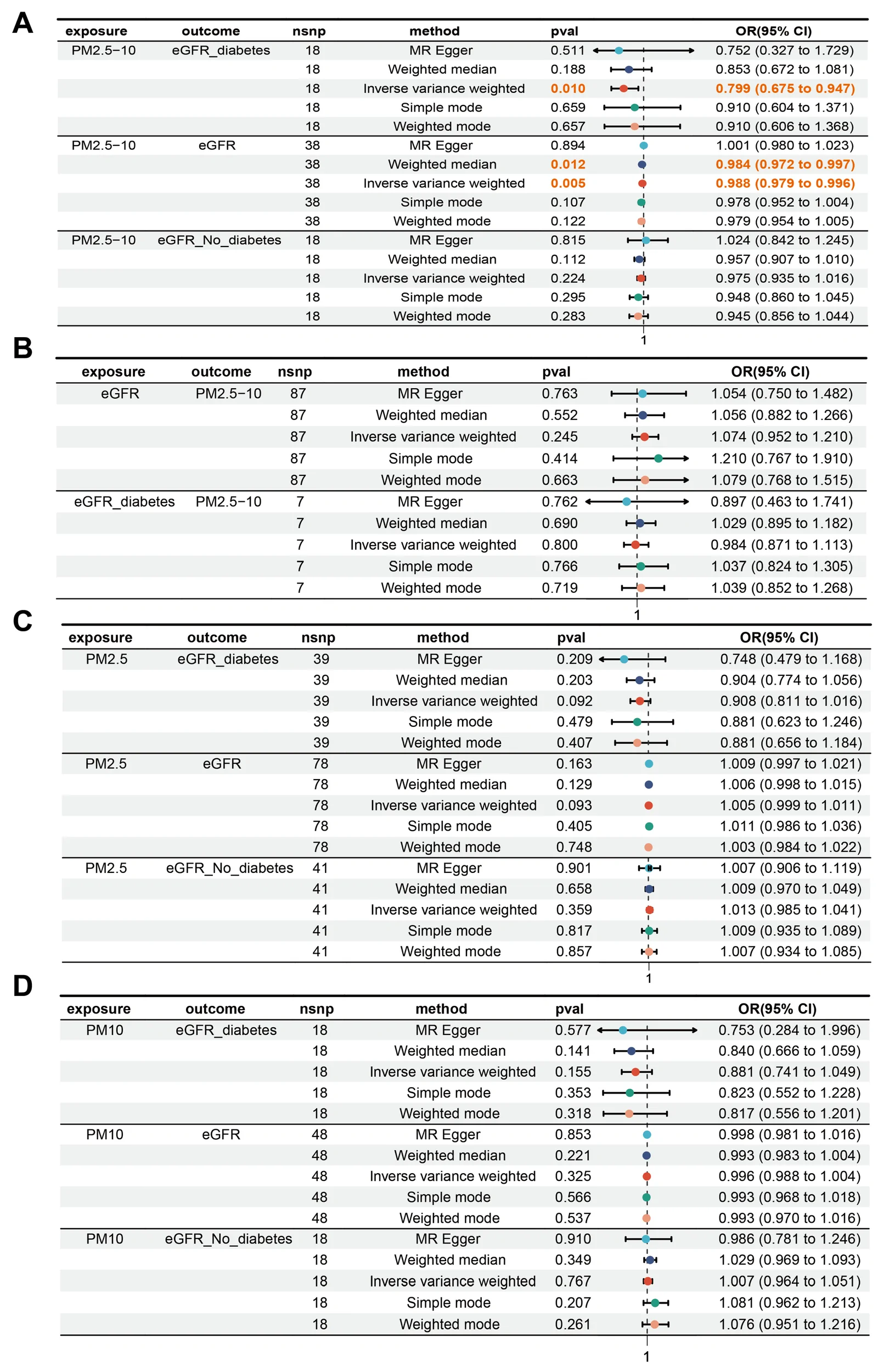
Bidirectional Mendelian randomization analyses between PM exposure and renal function. (**A**) Forward MR analyses of PM2.5–10 exposure on eGFR in diabetic individuals (eGFR_diabetes), non-diabetic individuals (eGFR_No_diabetes), and overall eGFR. (**B**) Reverse MR analyses of renal function traits on PM2.5–10 exposure. (**C**) Forward MR analyses of PM2.5 exposure on eGFR in eGFR_diabetes, eGFR_No_diabetes, and overall eGFR. (**D**) Forward MR analyses of PM10 exposure on eGFR in eGFR_diabetes, eGFR_No_diabetes, and overall eGFR. Forest plots show odds ratios (ORs) and 95% confidence intervals (CIs) estimated using different MR methods. The orange color indicates features with statistical significance.

To investigate the potential causal relationship between PM exposure and renal function, we performed a bidirectional MR analysis. In the forward MR analysis, with PM2.5, PM2.5–10, and PM10 as exposures and eGFR as outcome, the IVW method indicated a significant association between PM2.5–10 exposure and decreased eGFR (OR = 0.988, 95% CI: 0.979–0.996, *p* = 0.005). The weighted median method yielded consistent results (OR = 0.984, 95% CI: 0.972–0.997, *p* = 0.012) (Figure 1A). Further analysis showed a significant association between PM2.5–10 and reduced eGFR in diabetic individuals (OR = 0.799, 95% CI: 0.675–0.947, *p* = 0.010), while no significant association was observed in non-diabetic individuals (Figure 1A). No significant associations were observed for PM2.5 (Figure 1C) or PM10 (Figure 1D).

In the reverse MR analysis, no significant associations were observed between eGFR and PM2.5–10 (Figure 1B). MR-Egger intercept tests and Cochran’s Q tests showed no evidence of horizontal pleiotropy or heterogeneity (Supplementary Tables S1 and S2). MR-PRESSO analysis showed no evidence of horizontal pleiotropy or outlier SNPs for the association between PM2.5–10 exposure and eGFR (MR-PRESSO *p* = 0.385) or eGFR_diabetes (MR-PRESSO *p* = 0.440) (Supplementary Table S3). Furthermore, none of the 41 PM2.5–10 instrumental SNPs showed significant associations with potential confounding traits, including BMI, smoking, alcohol consumption, educational attainment, PM2.5, or PM10 (*p* < 1 × 10^−5^), supporting the specificity of the selected genetic instruments (Supplementary Table S4). Detailed information on the included instrumental SNPs is provided in Supplementary Table S5.

### 2.2. Significant Overlap Between PM-Related Genes and DKD Differentially Expressed Genes, Mainly Involved in Inflammation and Metabolic Pathways

**Figure 2:**
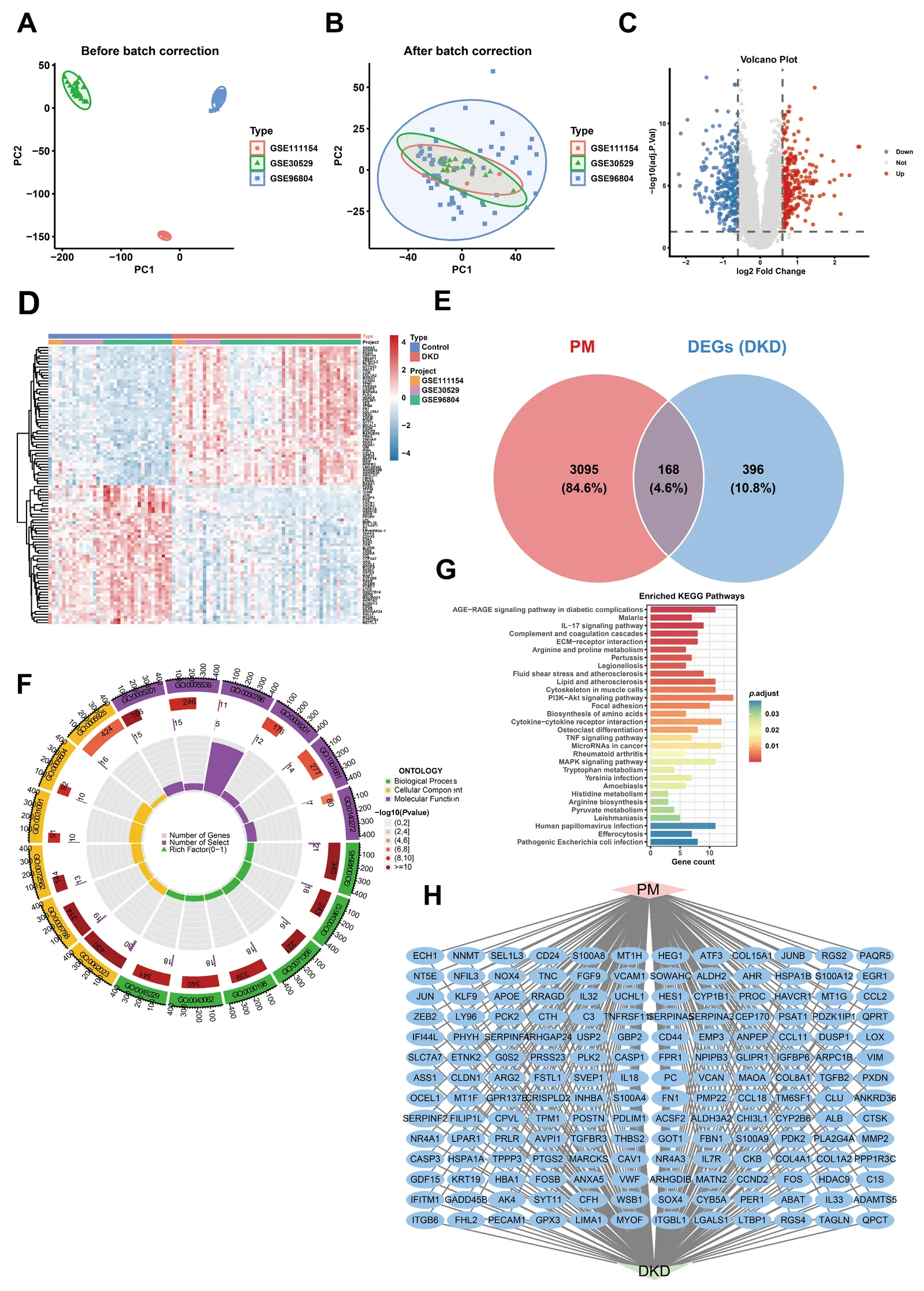
Identification and functional characterization of shared genes between PM exposure and DKD. Principal component analysis plots before (**A**) and after (**B**) batch correction of integrated datasets. (**C**) Volcano plot showing differentially expressed genes (DEGs) between DKD and control samples. (**D**) Heatmap of representative DEGs. (**E**) Venn diagram showing the overlap between PM-related genes and DKD DEGs, identifying 168 shared genes. (**F**) GO and KEGG (**G**) enrichment analyses of shared genes. (**H**) Interaction network of shared genes between PM and DKD.

To further explore the potential molecular mechanisms of PM exposure, we integrated multiple transcriptomic datasets to systematically analyze PM-related genes and DKD differentially expressed genes (DEGs). Batch effect correction was first performed across datasets. Principal component analysis (PCA) showed clear separation between datasets prior to correction (Figure 2A), while post-correction, samples from different datasets exhibited substantial overlap (Figure 2B), indicating the effective removal of batch effects.

Subsequent differential expression analysis identified a large number of significant DEGs, with upregulated and downregulated genes clearly distributed in the volcano plot (Figure 2C). Heatmap analysis further demonstrated that these genes effectively distinguished DKD samples from the controls (Figure 2D). Intersection analysis between PM-related genes and DKD DEGs identified 168 shared genes (Figure 2E), suggesting that these genes may represent potential molecular links between PM exposure and DKD.

GO enrichment analysis revealed that these shared genes were primarily involved in biological processes related to inflammatory response, extracellular matrix organization, oxidative stress, and immune regulation (Figure 2F). KEGG pathway analysis further demonstrated significant enrichment in the AGE-RAGE signaling pathway in diabetic complications, IL-17 signaling pathway, TNF signaling pathway, PI3K-Akt signaling pathway, and lipid and atherosclerosis pathway (Figure 2G), suggesting that PM exposure and DKD may share inflammatory, immune-related, and metabolic alterations. Although several infection-related KEGG pathways were also enriched, these pathways mainly reflected shared inflammatory and immune-related genes rather than specific infectious processes. Therefore, pathways directly associated with particulate matter exposure, diabetes-related renal injury, inflammation, and fibrosis were prioritized for further interpretation.

To further explore the potential functional interactions among PM-DKD shared genes, a protein–protein interaction (PPI) network was constructed based on these overlapping genes (Figure 2H). The network revealed potential interactions among multiple shared genes, suggesting that these genes may be involved in related biological processes associated with PM exposure and DKD. The observed interactions may provide a basis for further investigation of the biological roles and regulatory mechanisms of PM-DKD shared genes.

### 2.3. Machine Learning Identifies Robust PM-DKD Feature Genes with Strong Diagnostic Performance

**Figure 3:**
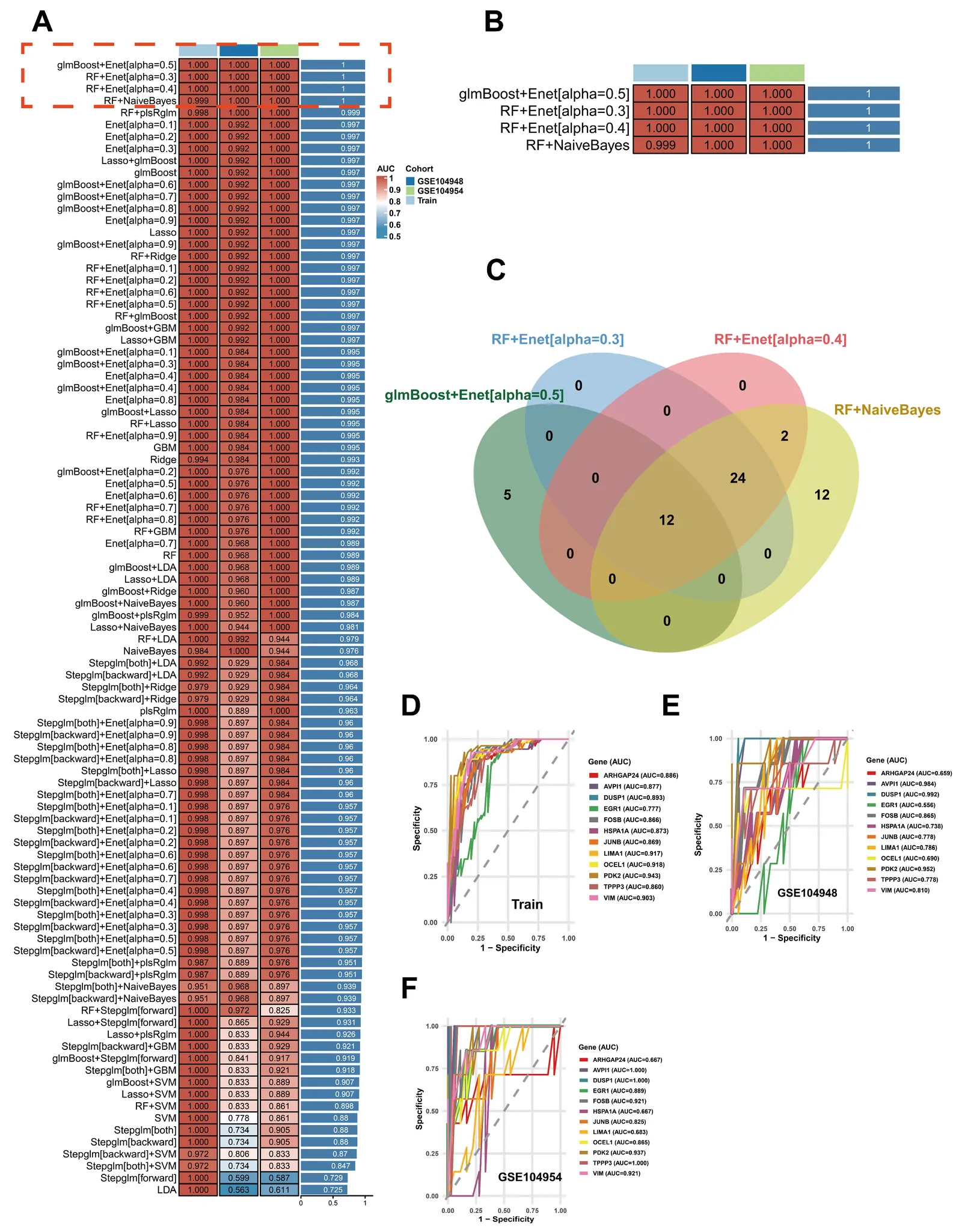
Identification of DKD diagnostic biomarkers using machine learning algorithms. (**A**) Heatmap of AUC values for 108 machine learning model combinations in the training and validation cohorts. (**B**) Four optimal machine learning models with the best diagnostic performance. (**C**) Venn diagram showing 12 overlapping feature genes identified by the four models. ROC curves of the 12 candidate biomarkers in the training cohort (**D**), GSE104948 cohort (**E**), and GSE104954 cohort (**F**). The dotted box highlights the four models with the highest area under the curve (AUC) values.

After identifying PM-DKD shared genes, multiple machine learning algorithms were applied to screen robust feature genes with potential diagnostic value. Several combined models demonstrated excellent classification performance in both the training and external validation cohorts, showing excellent discrimination performance (Figure 3A,B). Among these models, four top-performing algorithms, including glmBoost + Enet (α = 0.5), RF + Enet (α = 0.3), RF + Enet (α = 0.4), and RF + NaiveBayes, showed consistently high predictive performance, with AUC values approaching 1.0. Additional evaluation using accuracy, precision, recall, and F1-score further confirmed their reliable classification performance (Supplementary Table S7).

**Figure 4:**
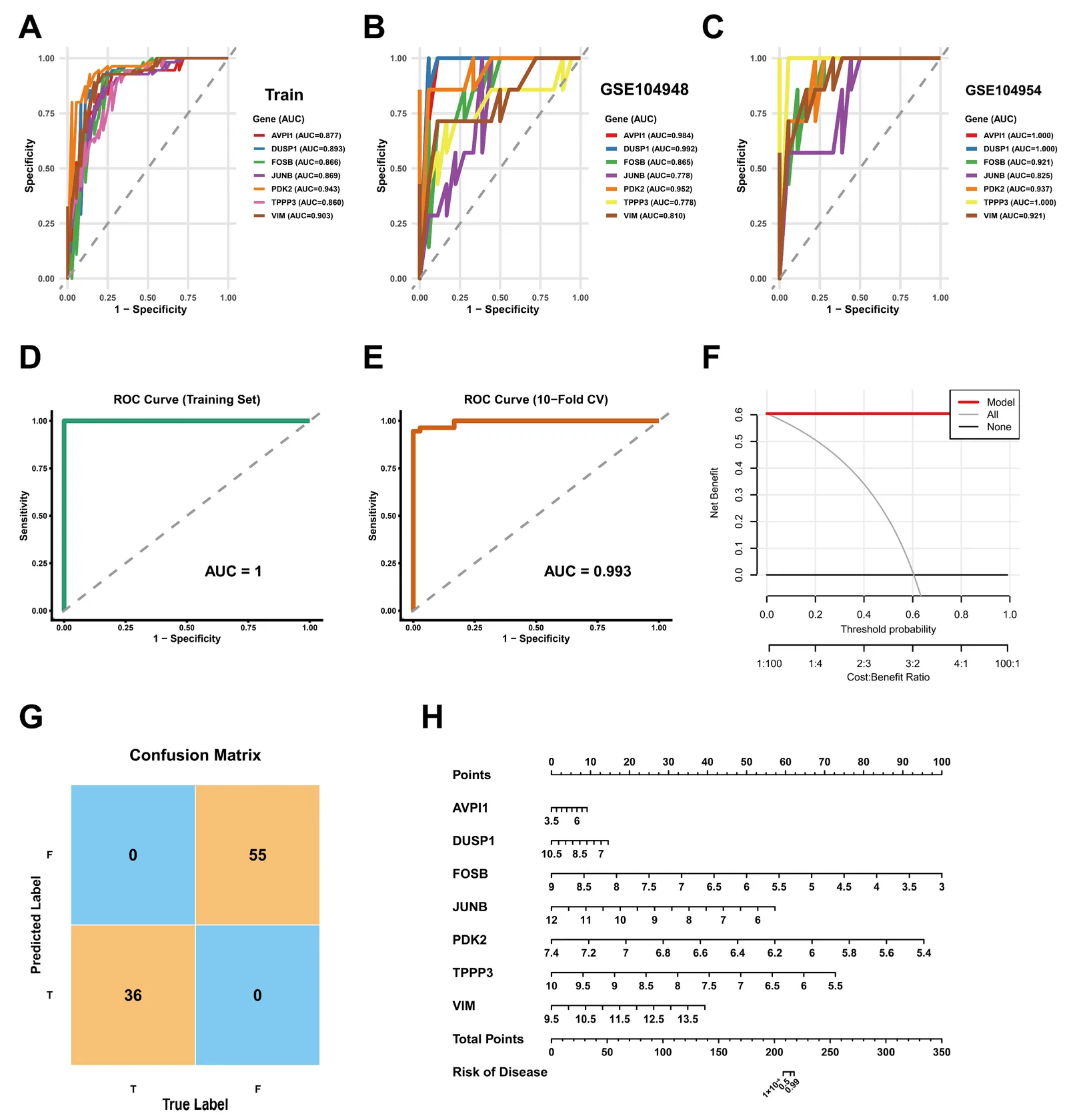
Validation and diagnostic evaluation of seven key biomarkers with AUC > 0.75. ROC curves of the seven biomarkers in the training cohort (**A**), GSE104948 cohort (**B**), and GSE104954 cohort (**C**). ROC analysis of the diagnostic model in the training set (**D**) and 10-fold cross-validation set (**E**). (**F**) Decision curve analysis of the diagnostic model. (**G**) Confusion matrix evaluating the classification performance of the model. (**H**) Nomogram constructed based on the seven key biomarkers for predicting DKD risk.

Venn analysis identified 12 overlapping feature genes among the optimal models, including ARHGAP24, AVPI1, DUSP1, EGR1, FOSB, HSPA1A, JUNB, LIMA1, OCLN, PDK2, TPPP3, and VIM (Figure 3C). ROC analysis further demonstrated that these genes exhibited favorable diagnostic performance across the training and external validation datasets, with most genes achieving AUC values greater than 0.75 (Figure 3D and Figure 4F).

Based on these findings, seven genes (AVPI1, DUSP1, FOSB, JUNB, PDK2, TPPP3, and VIM) with AUC values > 0.75 across all cohorts and stable diagnostic performance across datasets were retained for subsequent analyses. To evaluate the potential influence of the small-sample cohort GSE111154, sensitivity analysis was performed after excluding this cohort. Five genes (DUSP1, FOSB, JUNB, PDK2, and VIM) from the original seven-gene signature were consistently identified, indicating that these genes represent relatively stable components of the signature (Supplementary Figure S3A–C). A diagnostic model constructed using the seven-genes signature demonstrated excellent discriminative performance in the training cohort and remained highly stable after tenfold cross-validation (Figure 4A–E). Decision curve analysis suggested potential clinical utility (Figure 4F). In addition, confusion matrix analysis confirmed the ability of the model to distinguish DKD samples from the controls (Figure 4G). A nomogram integrating these feature genes was subsequently established to facilitate visualization of the predictive model (Figure 4H).

### 2.4. Feature Genes Exhibit Consistent Expression Patterns and Are Associated with Inflammatory and Metabolic Dysregulation in DKD

**Figure 5:**
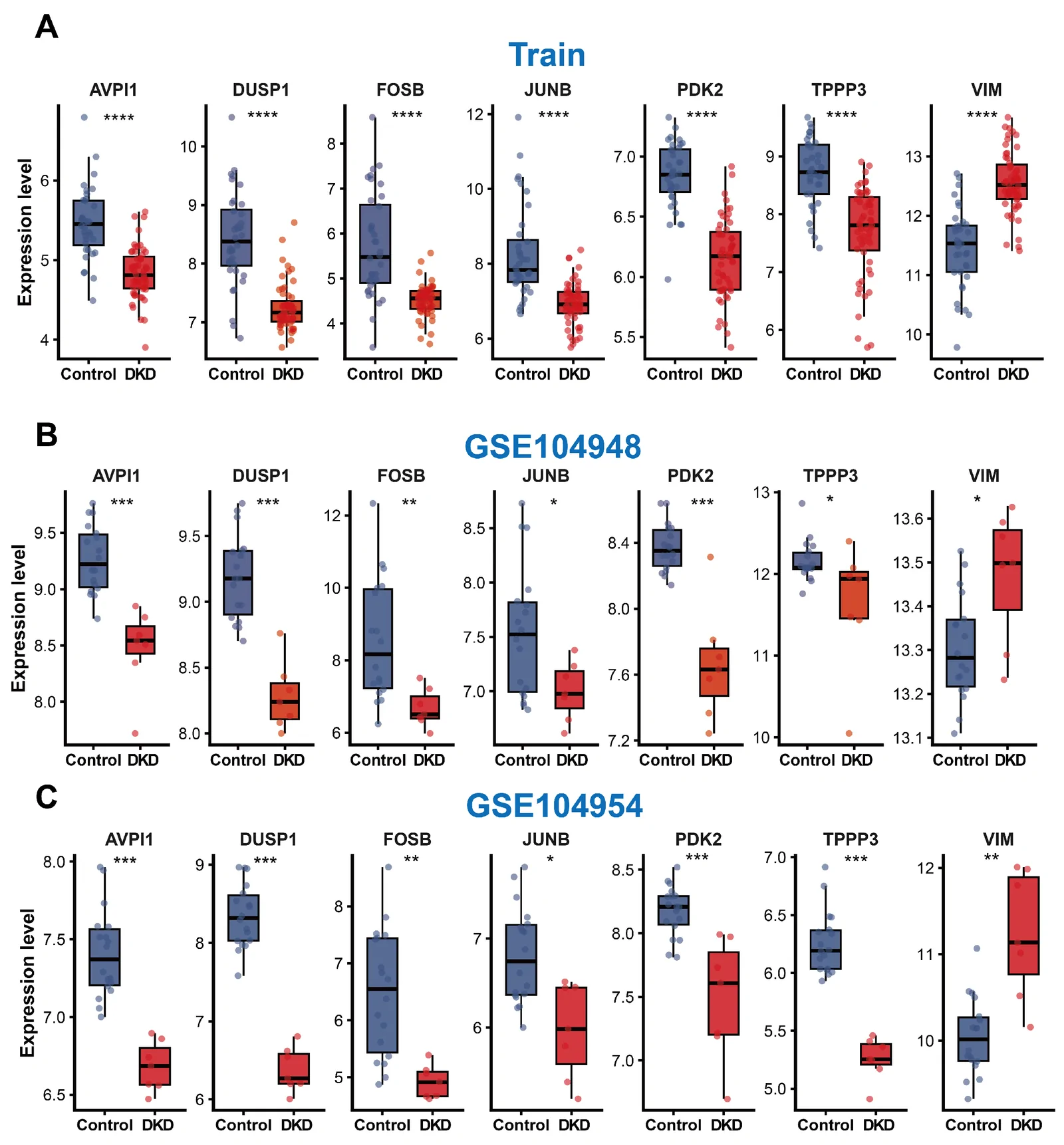
Expression validation of seven key biomarkers in DKD. Differential expression of AVPI1, DUSP1, FOSB, JUNB, PDK2, TPPP3, and VIM between the control and DKD samples in the training cohort (**A**), GSE104948 cohort (**B**), and GSE104954 cohort (**C**). Statistical significance was assessed using the Wilcoxon rank-sum test, and *p* values were considered significant at * *p* < 0.05, ** *p* < 0.01, *** *p* < 0.001, and **** *p* < 0.0001.

Expression patterns of AVPI1, DUSP1, FOSB, JUNB, PDK2, TPPP3, and VIM were further evaluated across the training and external validation cohorts. AVPI1, DUSP1, FOSB, JUNB, PDK2, and TPPP3 were consistently downregulated in the DKD samples, whereas VIM was markedly upregulated across all datasets (Figure 5A–C), suggesting stable transcriptional alterations associated with DKD progression.

Single-gene GSEA further revealed that these feature genes were mainly enriched in pathways related to inflammatory response, immune regulation, oxidative stress, and metabolic dysfunction, including chemokine signaling, cytokine–cytokine receptor interaction, Toll-like receptor signaling, JAK-STAT signaling, and peroxisome-related pathways (Supplementary Figure S1A–G). Among these genes, DUSP1, FOSB, and JUNB were more strongly associated with the inflammatory and stress-responsive signaling pathways, whereas VIM was preferentially enriched in extracellular matrix remodeling and fibrosis-related pathways. These findings suggest that the identified feature genes may be associated with DKD-related biological processes through inflammatory activation, metabolic dysregulation, and cellular injury processes.

### 2.5. ScRNA Analysis Reveals Distinct Cell-Type-Specific Expression of Feature Genes in DKD

**Figure 6:**
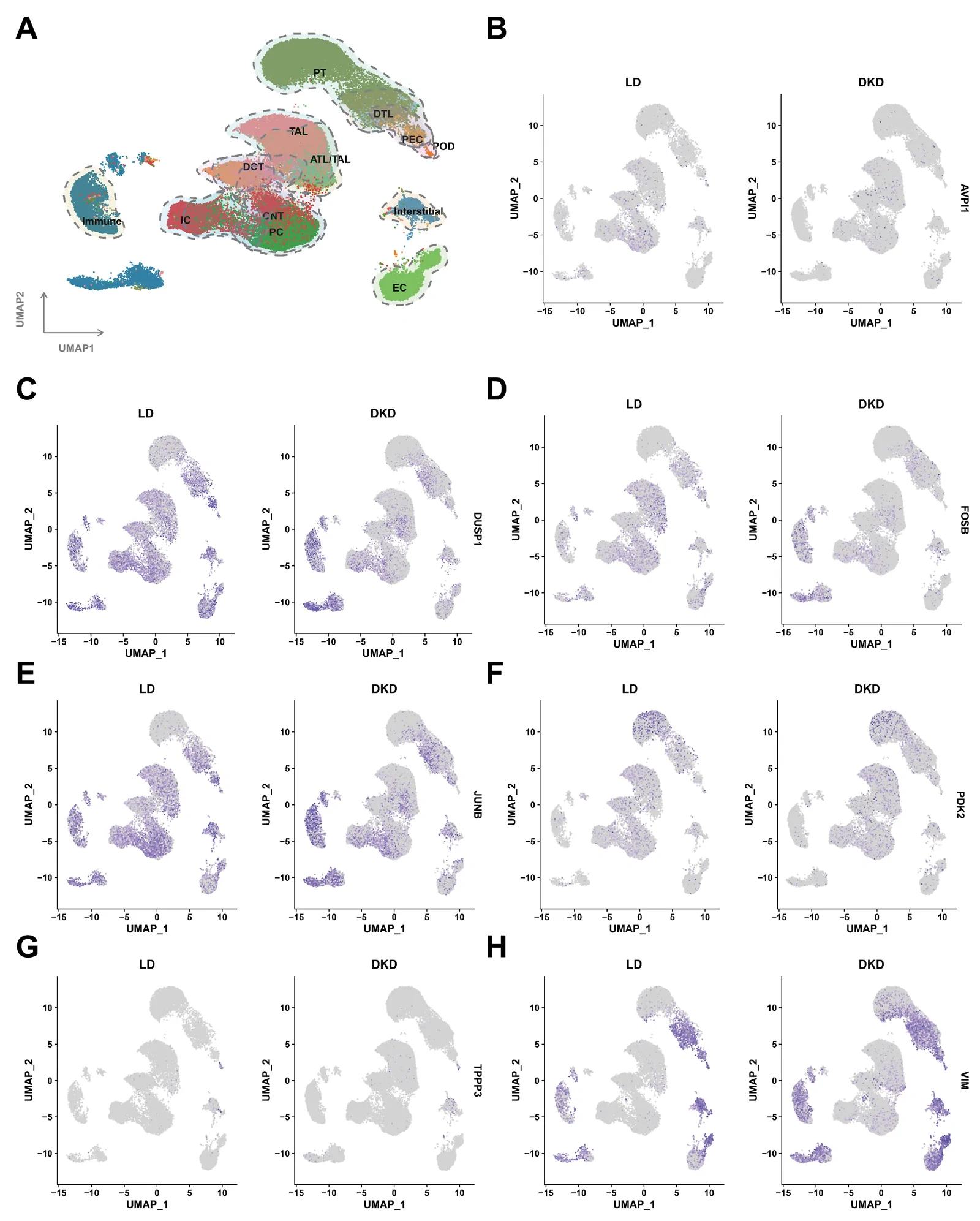
Single-cell expression patterns of feature genes in DKD. (**A**) UMAP plot displaying 13 renal cell populations identified by scRNA-Seq analysis. Feature plots showing the expression distribution of feature genes in the LD (living donor) and DKD samples, including AVPI1 (**B**), DUSP1 (**C**), FOSB (**D**), JUNB (**E**), PDK2 (**F**), TPPP3 (**G**), and VIM (**H**).

To further characterize the expression patterns of feature genes in different renal cell types, single-cell RNA sequencing data were analyzed. UMAP analysis clearly classified renal cells into multiple cell subpopulations, including PT, DTL, PEC, POD, TAL, ATL/TAL, DCT, CNT, PC, EC, IC, interstitial cells, and immune cells (Figure 6A).

Comparison of gene expression patterns between the LD and DKD groups revealed distinct cell-type-specific heterogeneity for AVPI1, DUSP1, FOSB, JUNB, PDK2, TPPP3, and VIM. AVPI1 exhibited generally low expression levels (Figure 6B). DUSP1, FOSB, and JUNB were primarily expressed in immune cells and renal tubular-related cells (Figure 6C–E). PDK2 was mainly enriched in renal tubular epithelial cells (Figure 6F), whereas TPPP3 showed overall low expression and was mainly detectable in podocytes (Figure 6G). In contrast, VIM exhibited relatively high expression in interstitial cells and certain tubular cell populations (Figure 6H).

To further validate these expression differences across cell types, grouped expression analyses were subsequently performed for each cell subpopulation. AVPI1 remained expressed at low levels overall (Supplementary Figures S2A and S4). DUSP1, FOSB, and JUNB were highly expressed in multiple renal tubular and immune cell populations, although their overall expression levels were reduced in the DKD group compared with the LD group (Supplementary Figure S2B–D). Meanwhile, PDK2 showed relatively low overall expression but was enriched in PT cells, suggesting a potential association with proximal tubular alterations in DKD (Supplementary Figure S2E). TPPP3 was mainly expressed in podocytes, suggesting a potential association with podocyte-related alterations in DKD (Supplementary Figure S2F). VIM was predominantly expressed in interstitial cells, PECs, and podocytes and was markedly elevated in the DKD group (Supplementary Figure S2G), further consistent with increased fibrotic remodeling and interstitial activation during DKD progression.

Collectively, these findings indicate that the differential expression of key genes across distinct renal cell populations reflects the complex cellular heterogeneity and potential molecular regulatory mechanisms underlying DKD.

### 2.6. Spatial Transcriptomic Analysis Further Validates the Regional Expression Patterns of Feature Genes in DKD

**Figure 7:**
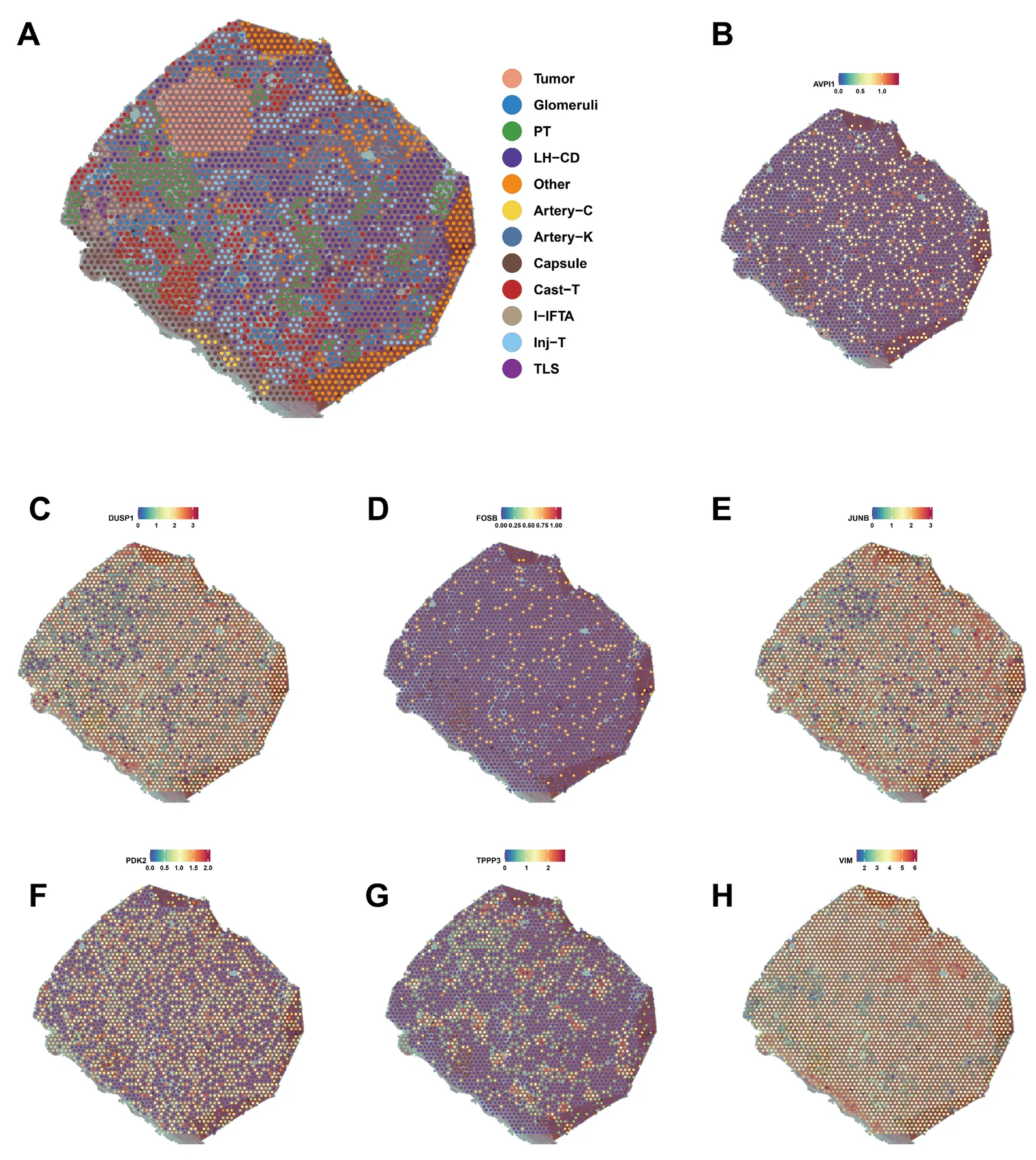
Spatial expression patterns of hub genes in kidney tissue. (**A**) Spatial distribution of annotated renal regions in spatial transcriptomics data. Spatial expression patterns of hub genes, including AVPI1 (**B**), DUSP1 (**C**), FOSB (**D**), JUNB (**E**), PDK2 (**F**), TPPP3 (**G**), and VIM (**H**).

To further validate the spatial distribution and histopathological relevance of the identified feature genes in DKD, spatial transcriptomic analysis was performed using a Visium-based renal cortex dataset from a patient with diabetic nephropathy. Histopathological annotation identified multiple renal compartments and lesion types, including glomeruli, proximal tubules, injured tubules, loop of Henle/collecting ducts, inflammatory fibrosis regions, tertiary lymphoid structures, arteries, and papillary tumor regions (Figure 7A).

Spatial expression analysis revealed distinct yet partially overlapping region-specific patterns of the seven feature genes across different renal pathological compartments. AVPI1 and FOSB showed generally low and diffuse expression across the tissue section (Figure 7B,D). In contrast, DUSP1, JUNB, and PDK2 showed relatively broad spatial distribution and were enriched in injured tubules, inflammatory infiltration areas, and fibrotic regions (Figure 7C,E,F), suggesting that these genes may be associated with stress-related and inflammatory processes in the diseased renal microenvironment. Notably, TPPP3 showed a more restricted spatial pattern and was preferentially enriched in glomerular regions (Figure 7G), consistent with the single-cell transcriptomic results. VIM was widely and markedly upregulated across fibrotic and injured regions (Figure 7H), suggesting a potential association with mesenchymal activation and interstitial fibrotic remodeling in DKD.

Overall, spatial transcriptomic analysis further confirmed the region-specific expression consistency of the identified feature genes and indicated that these genes show region-specific expression patterns associated with tubular injury, inflammatory activation, and fibrotic remodeling during DKD progression.

### 2.7. In Silico Knockout Analysis Suggests Potential Associations Between Feature Genes and Mitochondrial Metabolism and Oxidative Stress-Related Pathways

**Figure 8:**
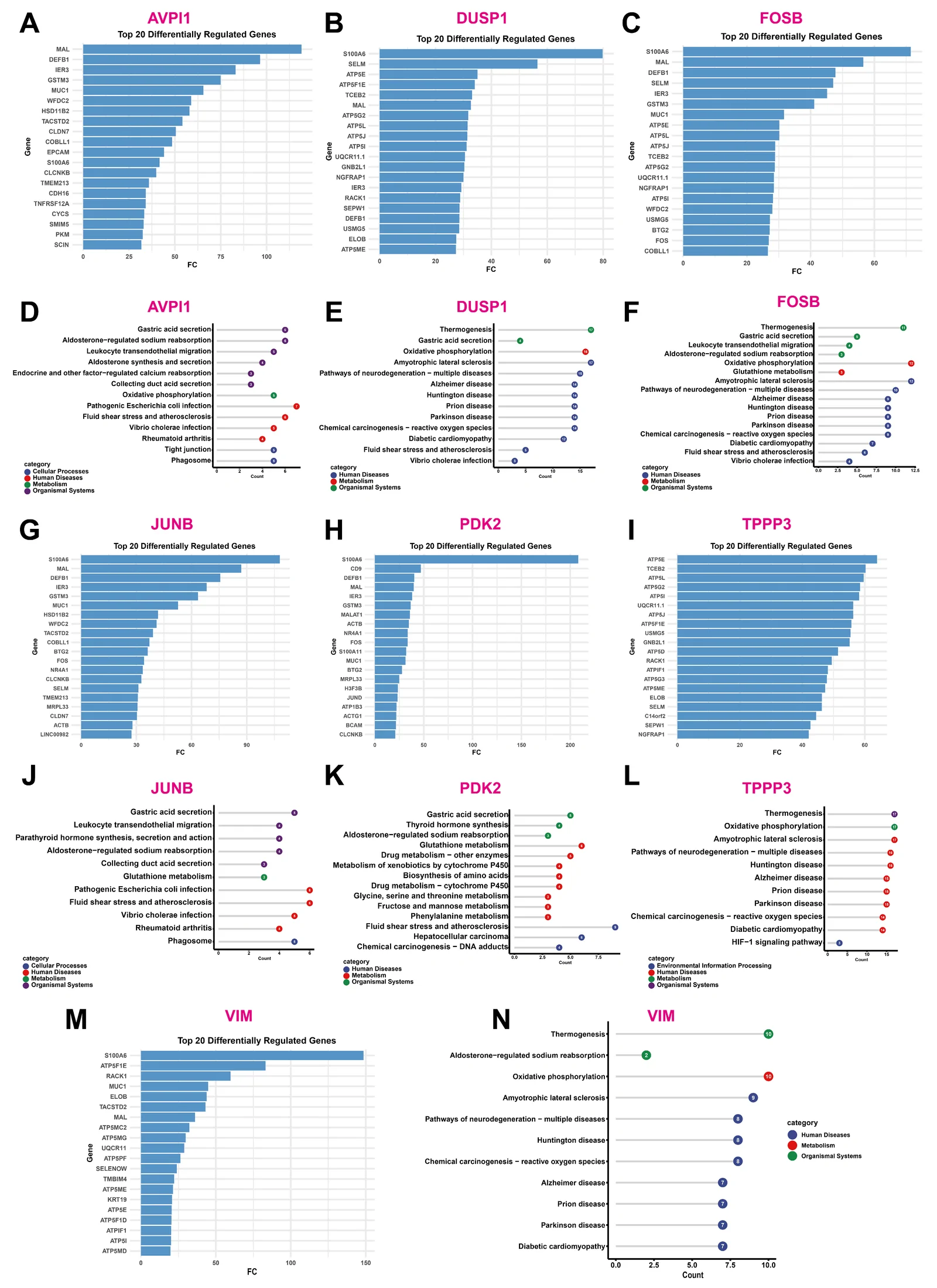
Functional characterization following in silico knockout of feature genes. Top 20 differentially regulated genes identified after in silico knockout of AVPI1 (**A**), DUSP1 (**B**), FOSB (**C**), JUNB (**G**), PDK2 (**H**), TPPP3 (**I**), and VIM (**M**). KEGG pathway enrichment analysis following virtual knockout of AVPI1 (**D**), DUSP1 (**E**), FOSB (**F**), JUNB (**J**), PDK2 (**K**), TPPP3 (**L**), and VIM (**N**), respectively.

In silico knockout analyses were subsequently performed to investigate the potential biological functions of the identified feature genes in DKD. Knockout of AVPI1, DUSP1, FOSB, and JUNB altered the expression of multiple renal tubular function-related genes, including MAL, DEFB1, MUC1, and ATP5 family genes (Figure 8A–C,G), suggesting potential involvement in tubular homeostasis and stress regulation. In addition, PDK2 and VIM knockout significantly affected oxidative phosphorylation-related genes, including ATP5F1E, ATP5MC3, and UQCR11 (Figure 8H,M), indicating possible roles in mitochondrial metabolic reprogramming.

KEGG enrichment analysis further demonstrated significant enrichment in oxidative phosphorylation, glutathione metabolism, thermogenesis, and aldosterone-regulated sodium reabsorption pathways (Figure 8D–F,J–L,N). Interestingly, several altered pathways overlapped with neurodegeneration-related signatures, suggesting shared mechanisms involving mitochondrial dysfunction and oxidative stress.

Moreover, TPPP3 and VIM knockout were associated with HIF-1 signaling and oxidative stress-related pathways, implying potential involvement in hypoxic injury and fibrotic remodeling during DKD progression.

### 2.8. VIM Represents a Potential Candidate Molecule in PM-Related DKD, with Sanguinarine as a Candidate Compound

Potential compounds associated with the identified feature genes were further explored through the analysis of traditional Chinese medicine (TCM)-derived active ingredients. Among the seven feature genes, VIM showed the most prominent association with multiple bioactive compounds in the database analysis, suggesting that VIM may represent a potential candidate molecule involved in PM-related DKD.

**Figure 9:**
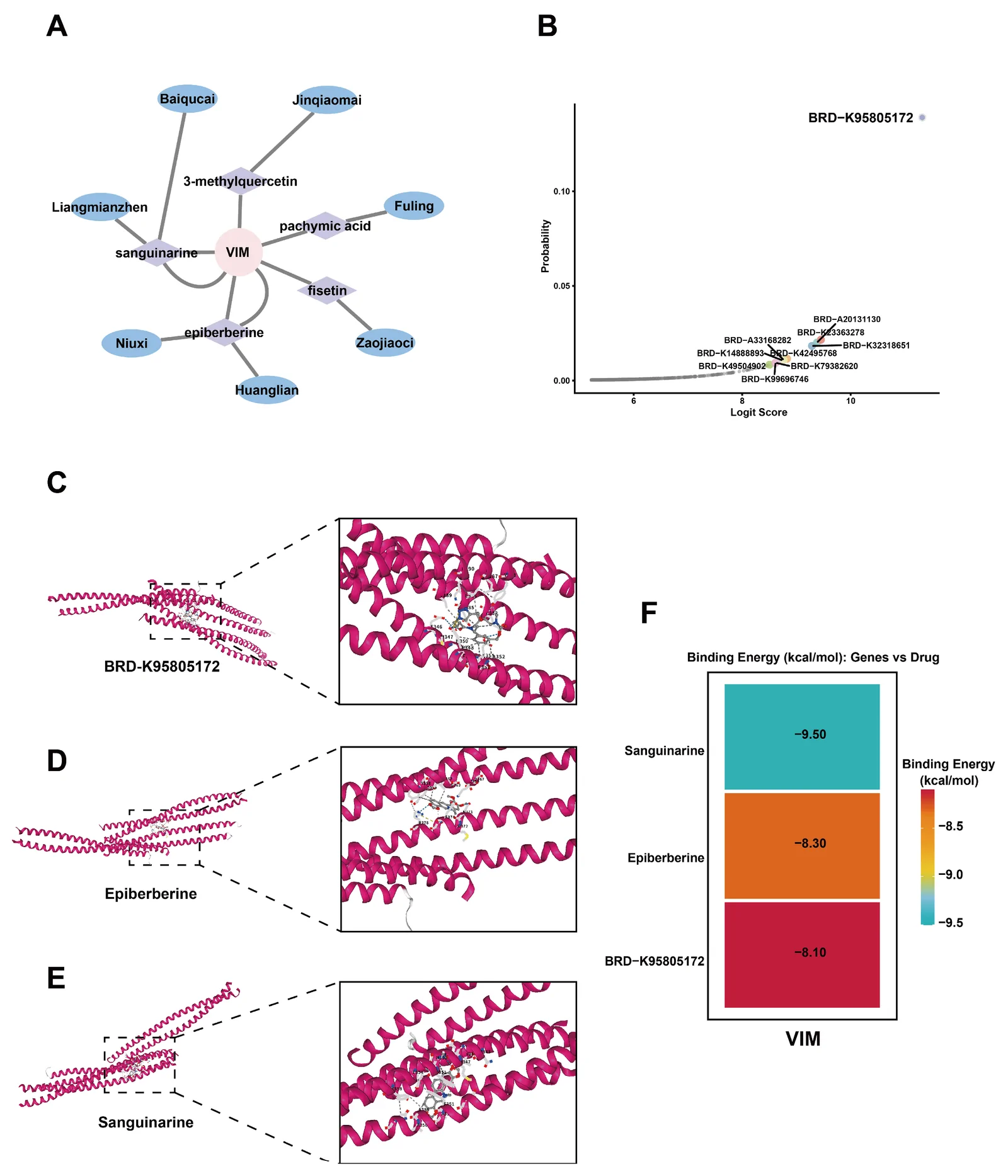
TCM compounds targeting VIM and molecular docking analysis. (**A**) “TCM–active ingredient–VIM” network showing VIM interactions with multiple compounds; epiberberine and sanguinarine show high connectivity. (**B**) DrugReflector analysis identifying BRD-K95805172 as the top candidate based on Logit score and prediction probability. Molecular docking of VIM with BRD-K95805172 (**C**), epiberberine (**D**), and sanguinarine (**E**), showing stable binding to the VIM active site. (**F**) Binding energies of the three compounds with VIM, with sanguinarine exhibiting the strongest affinity (−9.50 kcal/mol).

Network analysis identified several candidate compounds potentially associated with VIM, including sanguinarine, epiberberine, fisetin, 3-methylquercetin, and pachymic acid (Figure 9A). DrugReflector analysis further identified BRD-K95805172 as a candidate small-molecule compound with a relatively high prediction probability (Figure 9B). In addition, independent evidence from the Comparative Toxicogenomics Database (CTD) supported the potential association between sanguinarine and VIM, which is summarized in Supplementary Table S8.

Molecular docking analysis was subsequently performed to explore the potential interactions between VIM protein and candidate compounds, including BRD-K95805172, epiberberine, and sanguinarine (Figure 9C–E). Among these compounds, sanguinarine showed the lowest predicted binding energy (−9.50 kcal/mol), followed by epiberberine (−8.30 kcal/mol) and BRD-K95805172 (−8.10 kcal/mol) (Figure 9F), indicating relatively favorable predicted interactions with VIM.

Collectively, these findings suggest that VIM may represent a potential molecular candidate associated with PM-related DKD, and sanguinarine may warrant further investigation as a candidate compound. However, these database-based predictions and molecular docking results require further experimental validation.

## 3. Discussion

In this study, we systematically investigated the association between particulate matter (PM) exposure and DKD using a multi-method approach, including Mendelian randomization, transcriptomic analysis, and downstream bioinformatics analyses. PM2.5–10 exposure was genetically associated with reduced renal function, with a more pronounced association observed in individuals with diabetes, suggesting increased susceptibility of the diabetic kidney to environmental stressors. In addition, this study explored potential biological relevance by integrating PM-related genes with transcriptomic data to examine associated molecular pathways in DKD. The analysis further suggested that VIM may represent a potential candidate molecule, and sanguinarine may represent a candidate compound requiring further investigation.

Epidemiological studies consistently suggest that long-term PM exposure is associated with eGFR decline, albuminuria, and an increased risk of CKD [10,11,12,13,14]. Importantly, the adverse impact of PM2.5–10 on eGFR was amplified in individuals with diabetes. Chronic hyperglycemia in diabetes can exacerbate oxidative stress, sustain low-grade inflammation, and impair endothelial function [15,16,17,18]. PM exposure may further potentiate kidney injury in this high-risk context, although the precise molecular mechanisms remain unclear, and substantial heterogeneity across populations warrants further investigation. Beyond inflammation and oxidative stress, additional mechanisms may also contribute to PM-associated renal injury. Previous studies have suggested that PM exposure may disrupt the gut–kidney axis by altering intestinal microbiota composition, induce dysregulation of the renin–angiotensin system (RAS) and renal tubular injury, promote podocyte damage through PM-induced cellular stress responses, and contribute to renal injury through toxic components contained in PM, including heavy metals [19,20,21,22,23]. Although these mechanisms were not directly evaluated in the present study, they may represent complementary pathways linking environmental exposure to DKD progression and warrant further investigation.

Mechanistic analyses revealed that PM–DKD shared genes were predominantly enriched in inflammatory and immune-related pathways, including the AGE-RAGE, IL-17, TNF, and PI3K-Akt signaling pathways. These findings are consistent with previous evidence highlighting inflammation and oxidative stress as central drivers of DKD pathogenesis [24,25,26,27,28], suggesting that PM exposure may be associated with renal injury through inflammatory activation, oxidative stress-related processes, and metabolic alterations. Single-gene GSEA further demonstrated that AVPI1, DUSP1, FOSB, JUNB, PDK2, TPPP3, and VIM were mainly enriched in pathways associated with inflammatory response, immune regulation, and cellular stress, including chemokine signaling, cytokine–cytokine receptor interaction, Toll-like receptor signaling, and JAK-STAT signaling pathways. In addition, several genes were significantly associated with metabolic pathways, such as peroxisome, amino acid metabolism, and the cell adhesion molecule pathways, indicating that metabolic dysregulation may also contribute to PM-associated DKD progression. We identified seven candidate feature genes, AVPI1, DUSP1, FOSB, JUNB, PDK2, TPPP3, and VIM using machine learning. AVPI1 (arginine vasopressin-induced protein 1) encodes a protein annotated to participate in the regulation of the MAPK cascade according to Gene Ontology annotations. AVPI1 exhibited stable expression changes across datasets, although its functional role in kidney disease, particularly DKD, remains largely unexplored. DUSP1, FOSB, and JUNB are implicated in cellular stress and inflammatory regulation. DUSP1 has been reported to negatively regulate MAPK signaling [29], thereby mitigating inflammatory responses and conferring protection during tissue injury and oxidative stress. DUSP1 was downregulated in DKD, suggesting suppression of its protective regulatory function in disease states. FOSB and JUNB, members of the activator protein-1 (AP-1) transcription factor family, are key regulators of inflammatory and stress-responsive signaling pathways, modulating cytokine expression and cellular responses to oxidative stress [30,31]. The altered expression may reflect sustained chronic inflammation within the kidney. PDK2 also demonstrated potential relevance, consistent with the established role of the PDK family in modulating glycolysis and mitochondrial oxidative phosphorylation, processes integral to metabolic reprogramming [32,33]. VIM (vimentin) has been widely used as a mesenchymal marker of epithelial-to-mesenchymal transition (EMT), and its upregulation has been associated with extracellular matrix deposition and the progression of renal fibrosis [34,35]. In this study, VIM consistently exhibited elevated expression across multiple datasets, primarily enriched in interstitial and podocyte subpopulations. These results were further supported by spatial transcriptomic analysis, which demonstrated that DUSP1, JUNB, and PDK2 were preferentially enriched in injured tubules, inflammatory infiltration areas, and fibrotic regions, whereas VIM showed widespread expression across fibrotic and injured compartments. TPPP3 (tubulin polymerization-promoting protein family member 3) encodes a microtubule-associated protein involved in tubulin binding and microtubule bundle formation. In contrast, TPPP3 displayed a more restricted localization predominantly in glomerular regions, consistent with its podocyte-enriched expression observed in single-cell transcriptomic analysis. AVPI1 and FOSB showed relatively low and diffuse spatial expression across renal compartments. Collectively, these spatial patterns provide additional context for the cell-type specificity and regional heterogeneity of the identified genes within the diseased renal microenvironment.

In silico knockout analyses further suggested potential associations between these key genes and renal tubular epithelial homeostasis, mitochondrial function, and oxidative stress-related processes. Simulated knockout of AVPI1, DUSP1, FOSB, and JUNB was associated with altered expression of multiple renal tubular functional genes, including MAL, DEFB1, MUC1, and ATP5 family members, whereas PDK2 and VIM knockout showed potential associations with changes in oxidative phosphorylation-related genes, such as ATP5F1E, ATP5MC3, and UQCR11. These computational predictions suggest that mitochondrial energy metabolism and redox imbalance may represent potential downstream processes associated with DKD progression, although experimental validation is required. KEGG enrichment analyses additionally demonstrated significant enrichment in oxidative phosphorylation, glutathione metabolism, thermogenesis, and aldosterone-regulated sodium reabsorption pathways, suggesting that mitochondrial dysfunction and redox imbalance may represent important downstream mechanisms linking PM exposure to DKD progression. Interestingly, in silico knockout analyses indicated that multiple key genes are associated with oxidative phosphorylation and neurodegeneration-related pathways. These findings are consistent with previous reports suggesting that chronic kidney disease and neurodegenerative disorders, such as Alzheimer’s disease [36,37,38], may share common pathological mechanisms, including mitochondrial dysfunction, oxidative stress, and chronic inflammation, suggesting that mitochondrial dysfunction may represent a shared pathological feature across chronic degenerative conditions.

Finally, enrichment analysis of traditional Chinese medicine (TCM)-derived active compounds and molecular docking suggested that VIM was the only feature gene associated with TCM-derived bioactive compounds in our database analysis. Sanguinarine showed a predicted interaction with VIM based on molecular docking analysis, which is consistent with previous reports describing its anti-inflammatory properties [39] and potential effects on EMT-related processes [40]. However, its potential role in DKD requires further validation, including pharmacokinetic, bioavailability, safety, and functional studies. BRD-K95805172 also showed predicted interaction with VIM; however, its biological relevance and therapeutic potential remain to be determined.

Several limitations should be acknowledged. First, our study is primarily based on public databases and bioinformatics analyses, lacking in vivo or in vitro functional validation. Therefore, the identified genes, cellular localization patterns, and computational predictions should be considered hypothesis-generating rather than definitive mechanistic evidence. Second, molecular docking reflects theoretical ligand–target interactions and cannot directly predict in vivo biological or pharmacological effects, necessitating experimental confirmation. Third, although MR provides evidence supporting causal inference, potential horizontal pleiotropy and other limitations of genetic instruments cannot be completely excluded. In addition, the GWAS datasets used in this study were primarily derived from individuals of European ancestry, and the generalizability of our findings to other populations remains uncertain. Further studies involving diverse populations are needed to validate these associations. Furthermore, because the OpenGWAS database provides summary-level GWAS statistics rather than individual-level clinical data, detailed clinical characteristics, such as diabetes subtype (type 1 or type 2 diabetes) and CKD stage according to the KDIGO classification, were unavailable. Therefore, further detailed characterization of these clinical phenotypes was not possible in the current study. Finally, due to the lack of PM2.5–10-specific annotations, PM-related genes were used as proxies, which may limit particle size-specific interpretation. The significant association observed specifically for PM2.5–10 may reflect differences in particle composition, biological activity, exposure assessment accuracy, or statistical power among different PM fractions. PM2.5–10 contains relatively larger particles with distinct chemical components, which may contribute to different biological effects; however, further studies are needed to clarify fraction-specific mechanisms.

In conclusion, PM2.5–10 exposure may be associated with DKD, and PM-related gene analyses suggest the involvement of inflammatory, oxidative stress, metabolic, and mitochondrial-related pathways. Key genes, including VIM, were identified as potential candidate biomarkers, providing further insight into the relationship between environmental pollution and DKD.

## 4. Materials and Methods

### 4.1. Data Sources

Transcriptomic data for DKD were obtained from the Gene Expression Omnibus (GEO) database, with detailed information provided in Table 1. As PM2.5–10 does not represent an independently curated exposure in the Comparative Toxicogenomics Database (CTD), no exposure-specific gene annotations are available. CTD has been widely used to curate gene–environment interactions for particulate matter exposure. Therefore, PM-related genes were used as exploratory candidates to investigate general molecular responses associated with particulate matter exposure rather than as PM2.5–10-specific molecular markers.

### 4.2. MR Analysis

MR was performed to evaluate the potential causal relationship between particulate matter exposure and renal function. Instrumental variables (single nucleotide polymorphisms, SNPs) were obtained from openGWAS databases. Exposure and outcome data are summarized in Table 2.

Instrumental variables were selected based on the following criteria: genome-wide significance threshold (*p* < 1 × 10^−5^), linkage disequilibrium (r^2^ < 0.001), and physical distance (>10,000 kb). Although the conventional genome-wide significance threshold (*p* < 5 × 10^−8^) was considered, this criterion resulted in an insufficient number of instrumental variables for the particulate matter traits analyzed. Therefore, a threshold of *p* < 1 × 10^−5^ was applied. F-statistic was calculated to assess the strength of the instrumental variables, and only SNPs with F > 10 were retained to avoid weak instrument bias.

The inverse variance weighted (IVW) method was used as the primary MR approach, supplemented by weighted median, MR-Egger regression, simple mode, and weighted mode methods. Sensitivity analyses were performed using the MR-Egger regression method, and heterogeneity was assessed using Cochran’s Q test to evaluate the robustness of the results and potential horizontal pleiotropy. MR-PRESSO analysis was additionally performed to detect potential horizontal pleiotropy and identify possible outlier SNPs. Potential pleiotropic associations of instrumental variables with confounding traits, including BMI, smoking, alcohol consumption, educational attainment, PM2.5, and PM10, were evaluated. In the reverse MR analysis, estimated glomerular filtration rate (eGFR) was treated as the exposure, and particulate matter-related traits were treated as outcomes.

### 4.3. Transcriptomic Data Preprocessing and Differential Expression Analysis

For multiple GEO datasets, common genes shared among datasets were selected before data integration. Expression matrices were merged, and batch effects were corrected using the ComBat method implemented in the “sva” package. Principal component analysis (PCA) was performed to evaluate the effectiveness of batch correction. Differential expression analysis between the DKD and control groups was performed using the “limma” package based on a linear model framework. The empirical Bayes method was applied to moderate the variance estimates, and differentially expressed genes were identified using the criteria of |log2FC| > 0.6 and adjusted *p* value (FDR) < 0.05.

### 4.4. Intersection Gene Selection and Functional Enrichment Analysis

PM-related genes from CTD were intersected with DKD differentially expressed genes (DEGs) to obtain a shared gene set. Gene Ontology (GO) and Kyoto Encyclopedia of Genes and Genomes (KEGG) pathway enrichment analyses were performed using the ‘clusterProfiler’ package. A significance threshold of *p* < 0.05 was applied with multiple testing correction. Pathways related to inflammation, immune regulation, and metabolism were prioritized for interpretation.

### 4.5. Machine Learning Feature Selection and Model Construction

Candidate genes were used to construct multiple machine learning models, including random forest (RF), elastic net regression (Enet), gradient boosting model (glmBoost), Naive Bayes, and their combinations, totaling 108 methods. Models were trained on the training set and validated in independent external cohorts.

Model performance was evaluated using receiver operating characteristic (ROC) curves and area under the curve (AUC). Tenfold cross-validation was performed using the createFolds function in the caret package to evaluate model stability. The dataset was randomly divided into 10 subsets, with each subset sequentially used as the validation set while the remaining nine subsets were used for model training. The predicted probabilities from each fold were combined to generate cross-validated ROC curves and calculate AUC values. Stable feature genes were identified through intersection analysis among high-performing models. The selected genes were further evaluated using single-gene ROC analysis to assess their individual diagnostic potential. A multivariable logistic regression model based on the final seven-gene signature was subsequently constructed using the rms package for diagnostic prediction.

### 4.6. scRNA-Seq Analysis

DKD-related scRNA-Seq data were obtained from the Kidney Precision Medicine Project (KPMP)-annotated projects. The processed single-cell dataset (521c5b34-3dd0-4871-8064-61d3e3f1775a_PREMIERE_Alldatasets_08132021.h5Seurat) with cell-type annotations generated by the KPMP consortium was downloaded from the KPMP Atlas Explorer v1.3 dataset repository (DOI: 10.48698/92nk-e805) and used for downstream analyses. Since the dataset had undergone preprocessing, quality control, normalization, and cell-type annotation by the original investigators, these procedures were not repeated in the present study. Subsequent visualization and downstream analyses were performed in R (version 4.5.1).

### 4.7. Single-Gene GSEA Analysis

Gene set enrichment analysis (GSEA) was performed to explore the potential biological functions of feature genes. Samples were divided into high- and low-expression groups based on median gene expression. Pathway enrichment analysis was conducted using the MSigDB database, with significance thresholds set at *p* < 0.05 and FDR < 0.25.

### 4.8. Spatial Transcriptomic Analysis

Spatial transcriptomic data from GSE261545 were analyzed following the computational workflow described in a previously published study, with appropriate modifications according to the characteristics of the dataset and the objectives of the present study [41]. The dataset was generated using the 10× Genomics Visium platform from the renal cortex tissue of a patient with diabetic and hypertensive nephropathy. SpatialFeaturePlot was used to visualize the spatial expression patterns of AVPI1, DUSP1, FOSB, JUNB, PDK2, TPPP3, and VIM in different renal pathological regions.

### 4.9. In Silico Knockout Analysis

In silico knockout analysis was performed using the ‘scTenifoldKnk’ package to investigate the potential regulatory effects of candidate feature genes. Single-cell transcriptomic data from the DKD samples were used for the analysis. Highly variable genes were identified using the VST method, and the top 10,000 highly variable genes together with the target gene were used as input for the virtual knockout analysis. The scTenifoldKnk algorithm was then applied to simulate the perturbation of each target gene and identify genes with differential regulatory responses. Genes with an adjusted *p* value < 0.05 were considered significantly affected by the virtual knockout and were subsequently subjected to functional enrichment analysis.

### 4.10. Candidate Drug Screening and Molecular Docking

The ingredients of traditional Chinese medicine and their corresponding targets were retrieved from the Traditional Chinese Medicine Systems Pharmacology Database (TCMSP) to construct regulatory networks and identify potential bioactive compounds. DrugReflector (version 1.0.0) was used to predict potential small-molecule drugs. In addition, the Comparative Toxicogenomics Database (CTD) was used for independent validation of compound–gene associations, particularly the potential relationship between sanguinarine and VIM.

The three-dimensional structure of the VIM protein was obtained from the Protein Data Bank (PDB ID: 1G4K). Molecular docking was performed using the CB-Dock2 online docking platform, which integrates cavity detection and molecular docking algorithms for the prediction of ligand–protein interactions. Candidate compounds, including sanguinarine, epiberberine, and BRD-K95805172, were docked with VIM, and the predicted binding energies were used to evaluate relative binding affinity. Since CB-Dock2 performs automated docking procedures, docking parameters including binding pocket identification and docking calculations were determined by the platform.

### 4.11. Statistical Analysis

All statistical analyses were performed in R. Continuous variables were compared using the Student’s *t*-test. Multiple comparisons were corrected using the FDR method. ROC curves were used to evaluate diagnostic performance, and decision curve analysis (DCA) was used to assess clinical net benefit. *p* < 0.05 was considered statistically significant.

## Figures and Tables

**Table 1:** DKD transcriptomic datasets from GEO.

ID	Platform	Total Sample	Control	DKD
GSE96804	GPL17586	61	20	41
GSE104948	GPL22945	25	18	7
GSE104954	GPL22945	25	18	7
GSE111154	GPL17586	8	4	4
GSE30529	GPL571	22	12	10

**Table 2:** Exposure and outcome data.

ID	Trait	Population	Sample_Size
ebi-a-GCST003373	Glomerular filtration rate in diabetics	European	11,522
ebi-a-GCST90103634	Estimated glomerular filtration rate	European	1,004,040
ebi-a-GCST003401	Glomerular filtration rate in non-diabetics	European	118,448
ukb-b-11312	PM2.5	European	423,796
ukb-b-18469	PM10	European	423,796
ukb-b-12963	PM2.5–10	European	423,796

## Data Availability

The datasets analyzed during the current study are available in the openGWAS and GEO databases. The R scripts used for data processing and analysis are available in a public repository (https://github.com/Jieerry/ML (accessed on 9 August 2026)).

## Supplementary Materials

The following supporting information can be downloaded at: https://www.mdpi.com/article/10.3390/ijms27177615/s1.
