# Supplementary material for: Particulate Matter Exposure and Diabetic Kidney Dysfunction: Insights from Integrated Transcriptomic and Bioinformatics Analyses
Source: Int J Mol Sci. 2026 Aug 25;27(17):7615. doi: 10.3390/ijms27177615 (PMC13566131; doi:10.3390/ijms27177615)
Supplement: Supplementary file 1 [file ijms-27-07615-s001.zip › ijms-4463280-supplementary.pdf]

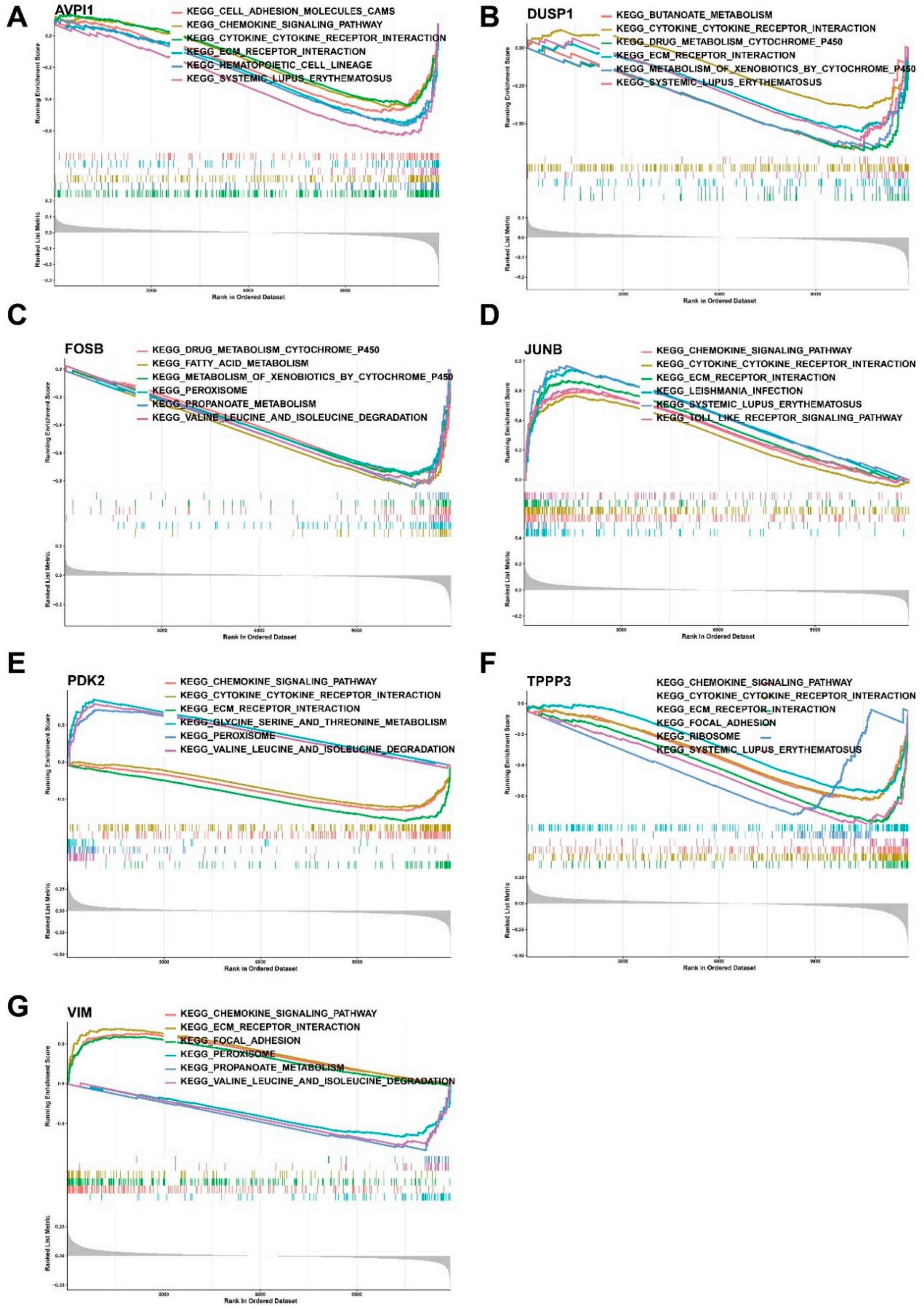

Supplementary Figure S1. GSEA enrichment analysis of seven feature genes.

GSEA showing enriched the top 6 KEGG pathways for AVPI1 (A), DUSP1 (B), FOSB (C), JUNB (D), PDK2 (E), TPPP3 (F), and VIM (G).

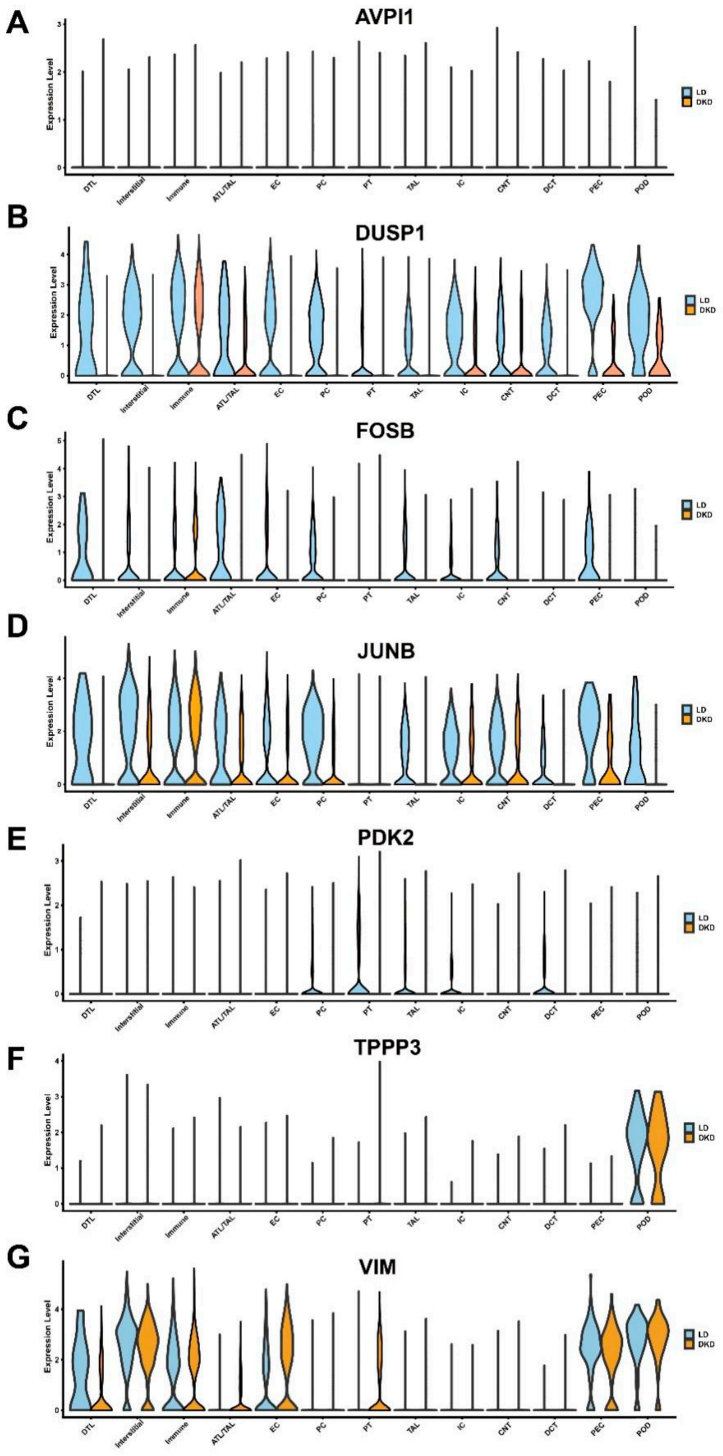

Supplementary Figure S2. Single-cell expression profiles of seven candidate genes.

Violin plots showing the expression levels of AVPI1 (A), DUSP1 (B), FOSB (C), JUNB (D), PDK2 (E), TPPP3 (F), and VIM (G) across different kidney cell types. LD, Living donor.

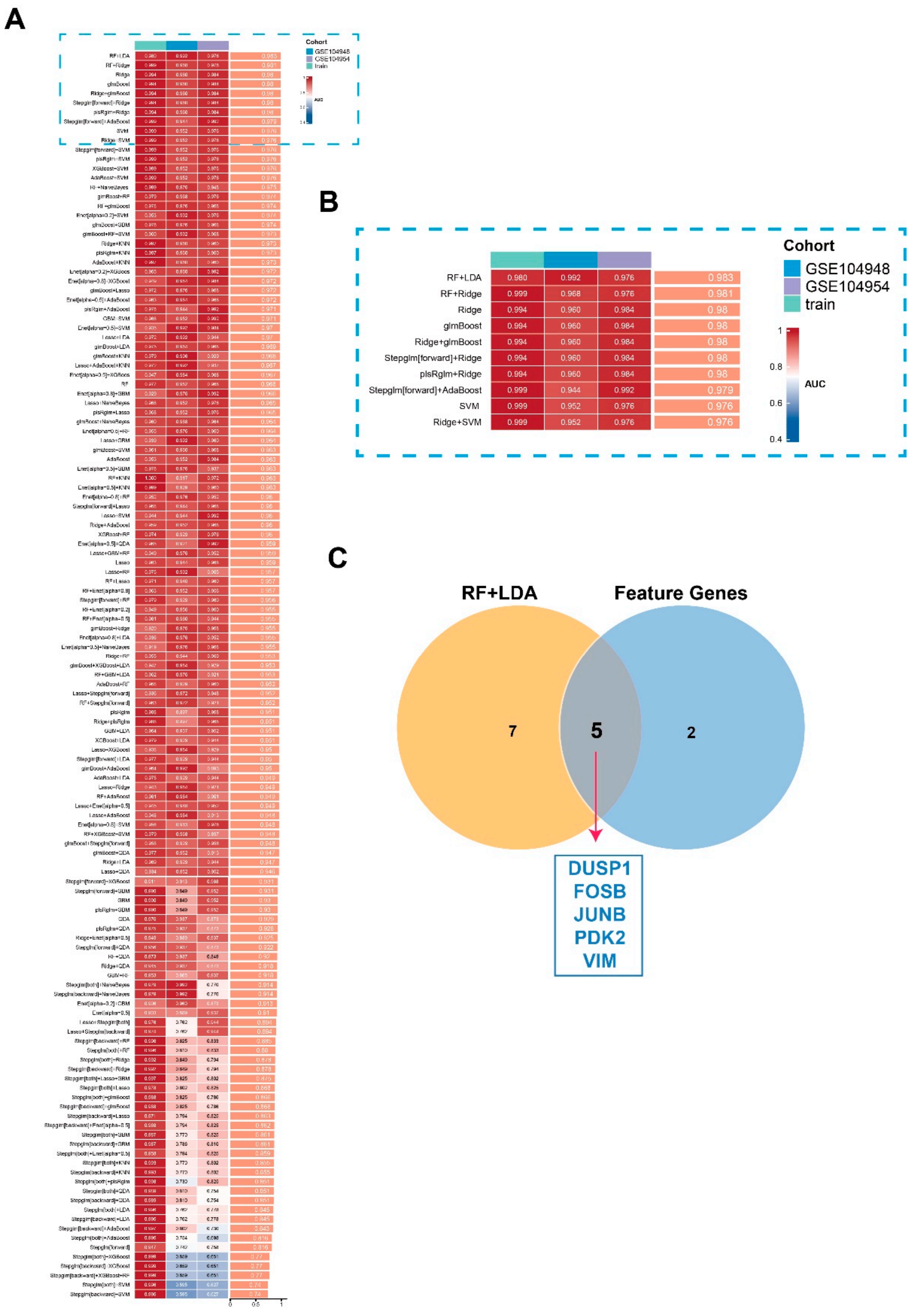

Supplementary Figure S3. Sensitivity analysis after excluding the GSE111154 cohort.

(A) AUC comparison of machine learning models after removal of GSE111154. (B) Performance of the top-ranked models after excluding GSE111154. (C) Overlap between the original seven-gene signature and genes identified after exclusion of GSE111154. Five genes (DUSP1, FOSB, JUNB, PDK2, and VIM) were consistently retained.

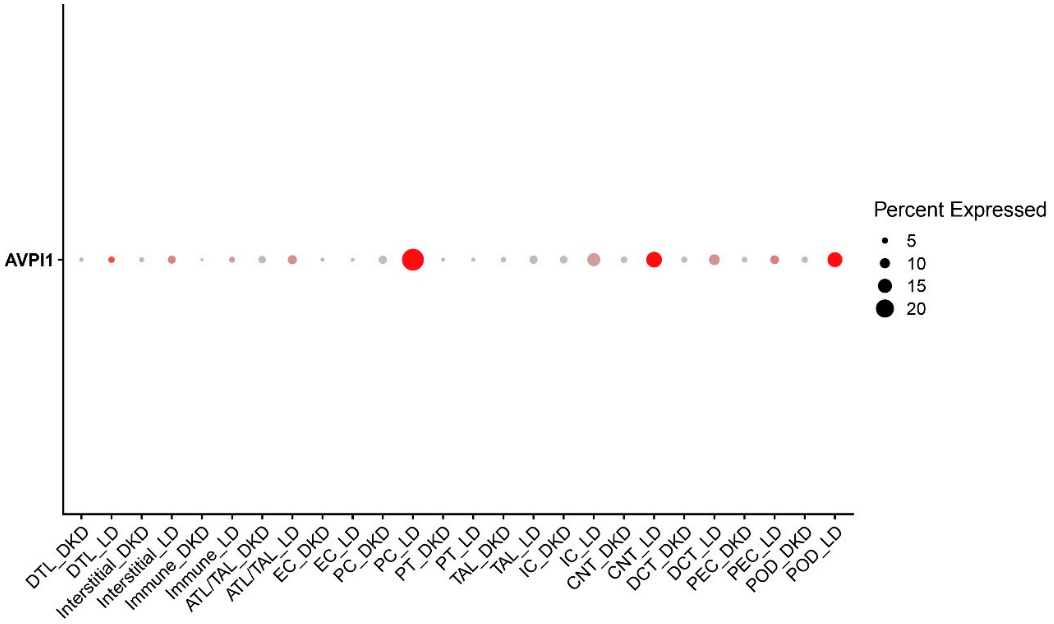

Supplementary Figure S4. Single-cell RNA-seq dot plot of AVPI1 expression across renal cell populations in DKD samples. LD, living donor; DKD, diabetic kidney disease.

Supplementary Table S1. MR-Egger intercept test for horizontal pleiotropy between PM2.5-10 and eGFR.

Exposure	Outcome	Egger_intercept	Se	Pval
PM2.5-10	eGFR_Diabete	0.0008	0.01	0.88
PM2.5-10	eGFR	-0.0002	0.0002	0.18

Supplementary Table S2. Heterogeneity test for the association between particulate matter exposure and eGFR using Cochran's Q statistics.

Exposure	Outcome	Method	Q	Q_df	Q_pval
PM2.5-10	eGFR_Diabete	MR Egger	17.72	16	0.34
PM2.5-10	eGFR_Diabete	Inverse variance weighted	17.74	17	0.41
PM2.5-10	eGFR	MR Egger	34.36	36	0.55
PM2.5-10	eGFR	Inverse variance weighted	36.25	37	0.50

Supplementary Table S3. MR-PRESSO sensitivity analysis for assessing horizontal pleiotropy.

Exposure	Outcome	SNPs	β	SE	P value	RSSobs	MR-PRESSO P	Outlier SNPs
PM2.5-10	eGFR	38	-0.0102	0.00434	0.024	42.043	0.385	No
PM2.5-10	eGFR_Diabete	18	-0.2239	0.08642	0.019	19.840	0.440	No

Supplementary Table S4. Evaluation of potential pleiotropic associations of instrumental variables with confounding traits.

Trait	GWAS ID	SNPs tested	Significant SNPs ($P < 1\times10^{-5}$)
BMI	ieu-b-40	41	0
Smoking	finn-b-SMOKING	41	0
Alcohol	ieu-b-4833	41	0
Educational attainment	ebi-a-GCST90029013	41	0

Supplementary Table S5. Characteristics of genetic instrumental variants for PM2.5–10 exposure.

id	chr	pos	SNPs	effect_allele	other_allele	eaf	beta	se	pval	samplesize	R2	F
ukb-b-12963	7	111672596	rs118101191	T	G	0.010103	0.0597911	0.0110633	6.50E-08	423796	6.89E-05	29.2
ukb-b-12963	14	103577789	rs11621531	A	G	0.063928	-0.0231281	0.00448732	2.50E-07	423796	6.27E-05	26.6
ukb-b-12963	8	18777006	rs1157546	C	T	0.053047	-0.0246035	0.00490067	5.20E-07	423796	5.95E-05	25.2
ukb-b-12963	7	105272307	rs1706918	A	G	0.098932	0.018349	0.00367669	6.00E-07	423796	5.88E-05	24.9
ukb-b-12963	19	22710669	rs12462492	T	G	0.257064	-0.0123914	0.00249623	6.90E-07	423796	5.81E-05	24.6

ukb-b-12963	6	148423453	rs9497937	A	C	0.232437	-0.0127713	0.00257727	7.20E-07	423796	5.79E-05	24.6
ukb-b-12963	5	37945741	rs78060907	A	C	0.020337	-0.0380539	0.00769756	7.70E-07	423796	5.77E-05	24.4
ukb-b-12963	15	40386911	rs10152521	C	T	0.002527	0.114394	0.0234312	1.00E-06	423796	5.62E-05	23.8
ukb-b-12963	5	3279667	rs116259145	A	C	0.031996	0.0301641	0.00618506	1.10E-06	423796	5.61E-05	23.8
ukb-b-12963	16	11041413	rs8051340	G	C	0.122584	0.0160734	0.00332586	1.30E-06	423796	5.51E-05	23.4
ukb-b-12963	3	116072052	rs71323440	T	C	0.114944	0.0162929	0.00340779	1.70E-06	423796	5.39E-05	22.9
ukb-b-12963	1	3592598	rs76170056	A	C	0.110936	0.0165077	0.003462	1.90E-06	423796	5.36E-05	22.7
ukb-b-12963	9	133798921	rs117389221	C	T	0.017253	-0.0402216	0.00850122	2.20E-06	423796	5.28E-05	22.4
ukb-b-12963	4	133858337	rs116816317	A	G	0.019771	0.0376491	0.00796504	2.30E-06	423796	5.27E-05	22.3
ukb-b-12963	4	110987693	rs138141967	T	G	0.01498	-0.0429378	0.00915495	2.70E-06	423796	5.19E-05	22.0
ukb-b-12963	7	35316123	rs17675316	G	A	0.021631	0.0369053	0.00788044	2.80E-06	423796	5.17E-05	21.9
ukb-b-12963	7	151623218	rs57048268	C	A	0.311303	-0.0110199	0.00236534	3.20E-06	423796	5.12E-05	21.7
ukb-b-12963	14	57123141	rs8006373	A	T	0.091464	-0.0175837	0.00377824	3.30E-06	423796	5.11E-05	21.7
ukb-b-12963	4	3468969	rs9997134	C	T	0.398721	-0.010473	0.002255	3.40E-06	423796	5.09E-05	21.6
ukb-b-	12	72900	rs6050	T	C	0.72	-0.011	0.0024	3.40	42379	5.09	21.

12963		322	27			9529	3937	5355	E-06	6	E-05	6
ukb-b-<br>12963	7	13421<br>408	rs1113<br>08789	A	T	0.05<br>6635	-0.021<br>9385	0.0047<br>2875	3.50<br>E-06	42379<br>6	5.08<br>E-05	21.<br>5
ukb-b-<br>12963	17	76358<br>269	rs6207<br>9137	C	T	0.10<br>7445	-0.017<br>3504	0.0037<br>4272	3.60<br>E-06	42379<br>6	5.07<br>E-05	21.<br>5
ukb-b-<br>12963	19	53268<br>990	rs1171<br>25329	G	C	0.00<br>8295	0.0551<br>672	0.0119<br>924	4.20<br>E-06	42379<br>6	4.99<br>E-05	21.<br>2
ukb-b-<br>12963	4	17806<br>9165	rs1312<br>5748	A	C	0.25<br>7878	0.0114<br>526	0.0025<br>016	4.70<br>E-06	42379<br>6	4.95<br>E-05	21.<br>0
ukb-b-<br>12963	20	12737<br>097	rs6078<br>746	G	A	0.35<br>7866	0.0103<br>55	0.0022<br>7683	5.40<br>E-06	42379<br>6	4.88<br>E-05	20.<br>7
ukb-b-<br>12963	18	74268<br>809	rs9956<br>831	T	C	0.01<br>4107	-0.041<br>8647	0.0092<br>0923	5.50<br>E-06	42379<br>6	4.88<br>E-05	20.<br>7
ukb-b-<br>12963	3	11330<br>0863	rs9868<br>243	C	T	0.19<br>5486	0.0124<br>51	0.0027<br>4417	5.70<br>E-06	42379<br>6	4.86<br>E-05	20.<br>6
ukb-b-<br>12963	9	93901<br>159	rs7033<br>430	G	T	0.76<br>3839	-0.011<br>7287	0.0025<br>8533	5.70<br>E-06	42379<br>6	4.86<br>E-05	20.<br>6
ukb-b-<br>12963	6	16381<br>395	rs4716<br>063	G	A	0.07<br>7556	-0.019<br>1355	0.0042<br>2184	5.80<br>E-06	42379<br>6	4.85<br>E-05	20.<br>5
ukb-b-<br>12963	1	36655<br>652	rs1748<br>399	T	C	0.57<br>361	0.0099<br>7495	0.0022<br>052	6.10<br>E-06	42379<br>6	4.83<br>E-05	20.<br>5
ukb-b-<br>12963	20	48404<br>077	rs6063<br>430	C	T	0.33<br>111	-0.011<br>1674	0.0024<br>7449	6.40<br>E-06	42379<br>6	4.81<br>E-05	20.<br>4
ukb-b-<br>12963	4	18941<br>9188	rs7456<br>1197	A	G	0.31<br>7562	-0.010<br>4975	0.0023<br>3259	6.80<br>E-06	42379<br>6	4.78<br>E-05	20.<br>3
ukb-b-<br>12963	12	78217<br>999	rs7905<br>0669	T	G	0.03<br>4066	0.0269<br>531	0.0060<br>0028	7.10<br>E-06	42379<br>6	4.76<br>E-05	20.<br>2
ukb-b-<br>12963	22	22401<br>006	rs4145<br>410	G	A	0.83<br>7657	-0.013<br>3621	0.0029<br>8339	7.50<br>E-06	42379<br>6	4.73<br>E-05	20.<br>1

ukb-b-12963	6	63055986	rs62414841	T	C	0.029361	0.0292893	0.00654516	7.60E-06	423796	4.72E-05	20.0
ukb-b-12963	15	92987146	rs74029037	T	C	0.037102	0.0256495	0.00574408	8.00E-06	423796	4.70E-05	19.9
ukb-b-12963	13	39169251	rs143921782	C	T	0.010493	0.0503784	0.0113484	9.00E-06	423796	4.65E-05	19.7
ukb-b-12963	5	32499805	rs72737245	G	A	0.23297	-0.0115499	0.00260226	9.10E-06	423796	4.65E-05	19.7
ukb-b-12963	8	132367285	rs10099257	T	C	0.229516	-0.0114712	0.00258987	9.50E-06	423796	4.63E-05	19.6
ukb-b-12963	9	16238128	rs7023300	A	T	0.338785	-0.0102891	0.00232358	9.50E-06	423796	4.63E-05	19.6
ukb-b-12963	12	117175608	rs73220422	T	C	0.028414	0.0291877	0.00659592	9.60E-06	423796	4.62E-05	19.6

Supplementary Table S6. GSEA-based KEGG pathway enrichment analysis of the seven machine learning-derived feature genes.

GENE	KEGG	setSize	enrichmentScore	NES	pvalue	p.adjust	qvalue
AVPI1	KEGG_CYTOKINE_CYTOKINE_RECEPTOR_INTERACTION	235	-0.511703832	-2.156575204	1e-10	1.83e-08	1.49473684210526e-08
	KEGG_HEMATOPOIETIC_CELL_LINEAGE	87	-0.641570125	-2.336580305	1.74421563330861e-09	1.59595730447738e-07	1.30357168384117e-07
	KEGG_CHEMOKINE_SIGNALING_PATHWAY	167	-0.523082219	-2.107273931	1.41349947129632e-08	8.62234677490756e-07	7.04269912014308e-07
	KEGG_SYSTEMIC_LUPUS_ERYTHEMATOSUS	52	-0.700952441	-2.367611119	2.60766524283311e-08	1.19300684859615e-06	9.74443327585006e-07
	KEGG_ECM_RECEPTOR_INTERACTION	81	-0.625409887	-2.250370162	5.95509241870245e-08	2.1795638252451e-06	1.78025920727526e-06
	KEGG_CELL_ADHESION_MOLECULES_CAMS	118	-0.556293436	-2.165579647	1.49145165960344e-07	4.54892756179048e-06	3.71554623971383e-06
	KEGG_FOCAL_ADHESION	189	-0.47420541	-1.947266941	2.37747764747543e-07	6.21540584982863e-06	5.07671918709039e-06
DUS	KEGG_DRUG_METABOLISM_CYTOCHROME_P450	55	-0.676722677	-2.31467665	5.519708593447	1.0101066726009	7.8437964222677

P1					69e-08	3e-05	7e-06
	KEGG_METABOLISM_OF_XENOBIOTICS_BY_CYTOCHROME_P450	51	-0.654528298	-2.210954987	7.81011638605113e-07	7.14625649323679e-05	5.54929322166791e-05
	KEGG_ECM_RECEPTOR_INTERACTION	81	-0.553153479	-2.069231794	2.82537362949607e-06	0.00017234779139926	0.000133833487712972
	KEGG_SYSTEMIC_LUPUS_ERYTHEMATOSUS	52	-0.609665467	-2.06596865	1.89763836176464e-05	0.000868169550507323	0.000674160996942701
	KEGG_CYTOKINE_CYTOKINE_RECEPTOR_INTERACTION	235	-0.398173195	-1.730378785	2.6206103850679e-05	0.000959143400934853	0.000744805056808773
	KEGG_BUTANOATE_METABOLISM	33	-0.670017038	-2.07400299	3.41199990321251e-05	0.00104065997047981	0.000808105240234541
FOSB	KEGG_FATTY_ACID_METABOLISM	39	-0.834183836	-2.508694445	1e-10	9.15e-09	6.05263157894737e-09
	KEGG_PEROXISOME	70	-0.741678058	-2.502808185	1e-10	9.15e-09	6.05263157894737e-09
	KEGG_VALINE_LEUCINE_AND_ISOLEUCINE_DEGRADATION	40	-0.812696607	-2.445991529	1.74505884781018e-10	1.06448589716421e-08	7.04146552625161e-09
	KEGG_DRUG_METABOLISM_CYTOCHROME_P450	55	-0.759325559	-2.433503568	4.89379521755703e-10	2.23891131203234e-08	1.48101697373436e-08
	KEGG_METABOLISM_OF_XENOBIOTICS_BY_CYTOCHROME_P450	51	-0.760354481	-2.42393793	8.62970944574357e-10	3.15847365714215e-08	2.08929807633792e-08
	KEGG_PROPANOATE_METABOLISM	27	-0.837977454	-2.337660324	8.54912756869307e-09	2.60748390845139e-07	1.72482398315737e-07
JUNB	KEGG_LEISHMANIA_INFECTION	69	0.745790819481073	2.49826770740177	1e-10	4.575e-09	3e-09
	KEGG_SYSTEMIC_LUPUS_ERYTHEMATOSUS	52	0.772466244196527	2.45054928037998	1e-10	4.575e-09	3e-09
	KEGG_CHEMOKINE_SIGNALING_PATHWAY	167	0.598447567211377	2.2589519773116	1e-10	4.575e-09	3e-09
	KEGG_CYTOKINE_CYTOKINE_RECEPTOR_INTERACTION	235	0.570626896220644	2.25668479356726	1e-10	4.575e-09	3e-09
	KEGG_ECM_RECEPTOR_INTERACTION	81	0.67177657464081	2.3113548380307	1.05747047210064e-09	3.87034192788833e-08	2.53792913304153e-08
	KEGG_TOLL_LIKE_RECEPTOR_SIGNALING_PATHWAY	97	0.618821439094066	2.19824061915453	1.07029011739329e-08	3.26438485804953e-07	2.14058023478658e-07
PDK2	KEGG_ECM_RECEPTOR_INTERACTION	81	-0.753877781	-2.488451791	1e-10	2.81378237247163e-09	1.48903064864763e-09
	KEGG_VALINE_LEUCINE_AND_ISOLEUCINE_DEGRADATION	40	0.830548415046158	2.46450548705889	1.07631019712029e-10	2.81378237247163e-09	1.48903064864763e-09
	KEGG_PEROXISOME	70	0.752264662570661	2.45867949104616	1e-10	2.81378237247163e-09	1.48903064864763e-09
	KEGG_GLYCINE_SERINE_AND_THREONINE_METABOLISM	27	0.88781218184001	2.41186287078225	1e-10	2.81378237247163e-09	1.48903064864763e-09

	KEGG_CHEMOKINE_SIGNALING_PATHWAY	167	-0.610514461	-2.260505904	1e-10	2.81378237247163e-09	1.48903064864763e-09
	KEGG_CYTOKINE_CYTOKINE_RECEPTOR_INTERACTION	235	-0.568952606	-2.201807545	1e-10	2.81378237247163e-09	1.48903064864763e-09
TPPP3	KEGG_ECM_RECEPTOR_INTERACTION	81	-0.720058387	-2.441492828	1e-10	9.15e-09	6.94736842105263e-09
	KEGG_CYTOKINE_CYTOKINE_RECEPTOR_INTERACTION	235	-0.576674359	-2.246629116	1e-10	9.15e-09	6.94736842105263e-09
	KEGG_CHEMOKINE_SIGNALING_PATHWAY	167	-0.578575071	-2.185699899	1.77721942548504e-10	1.08410384954588e-08	8.23133207593073e-09
	KEGG_SYSTEMIC_LUPUS_ERYTHEMATOSUS	52	-0.742237108	-2.311303026	3.52063815374521e-09	1.61069195533843e-07	1.22295851656413e-07
	KEGG_RIBOSOME	74	-0.674720743	-2.269175939	1.16951263479365e-08	4.28041624334475e-07	3.25001405879498e-07
	KEGG_FOCAL_ADHESION	189	-0.529334162	-2.023003403	3.67759670616897e-08	1.12166699538154e-06	8.51653974060182e-07
VIM	KEGG_PEROXISOME	70	-0.661002621	-2.528757044	1e-10	6.1e-09	3.6140350877193e-09
	KEGG_CHEMOKINE_SIGNALING_PATHWAY	167	0.635765505861054	2.29927894009234	1e-10	6.1e-09	3.6140350877193e-09
	KEGG_FOCAL_ADHESION	189	0.598121124516346	2.17982244716002	1e-10	6.1e-09	3.6140350877193e-09
	KEGG_ECM_RECEPTOR_INTERACTION	81	0.696223559492993	2.26354173446416	2.6387309819347e-10	1.20721942423513e-08	7.15234976682301e-09
	KEGG_VALINE_LEUCINE_AND_ISOLEUCINE_DEGRADATION	40	-0.764163987	-2.587616885	4.84241476076302e-10	1.77232380243927e-08	1.05003941128124e-08
	KEGG_PROPANOATE_METABOLISM	27	-0.829060331	-2.543313862	7.2767417205608e-10	2.21940622477104e-08	1.31491999511888e-08

Supplementary Table S7. Performance evaluation of the four optimal machine learning models for DKD classification.

Model	Accuracy	Precision	Recall	F1-score	AUC
glmBoost+Enet (α=0.5)	0.929	0.873	1.000	0.932	1.000
RF+Enet (α=0.3)	0.922	0.863	1.000	0.926	1.000
RF+Enet (α=0.4)	0.936	0.885	1.000	0.939	1.000
RF+NaiveBayes	0.936	0.905	0.971	0.937	1.000

Supplementary Table S8. CTD-based evidence supporting the association between sanguinarine and VIM.

Compound	Compound ID	CAS Number	Target Gene	Interaction Type	Species	Effect	PMID
Sanguinarine	C005705	2447-54-3	VIM	mRNA/Protein	Homo sapiens	Decreases expression	37393519
